# Supplementary material for: Spatially resolved transcriptomics reveal the determinants of primary resistance to immunotherapy in NSCLC with mature tertiary lymphoid structures
Source: Cell Rep Med. 2025 Feb 4;6(2):101934. doi: 10.1016/j.xcrm.2025.101934 (PMC11866545; doi:10.1016/j.xcrm.2025.101934)
Supplement: Document S1. Figures S1–S6 and Tables S1–S3 and S7 [file mmc1.pdf]

Cell Reports Medicine, Volume *6*

Supplemental information

Spatially resolved transcriptomics reveal the determinants of primary resistance to immunotherapy in NSCLC with mature tertiary lymphoid structures

Florent Peyraud, Jean-Philippe Guégan, Christophe Rey, Oren Lara, Ophélie Odin, Marie Del Castillo, Lucile Vanhersecke, Jean-Michel Coindre, Emma Clot, Maxime Brunet, Thomas Grellety, Angélique Tasseel, Sylvestre Le Moulec, Robert J. Johnston, Alban Bessede, and Antoine Italiano

SUPPLEMENTAL INFORMATION

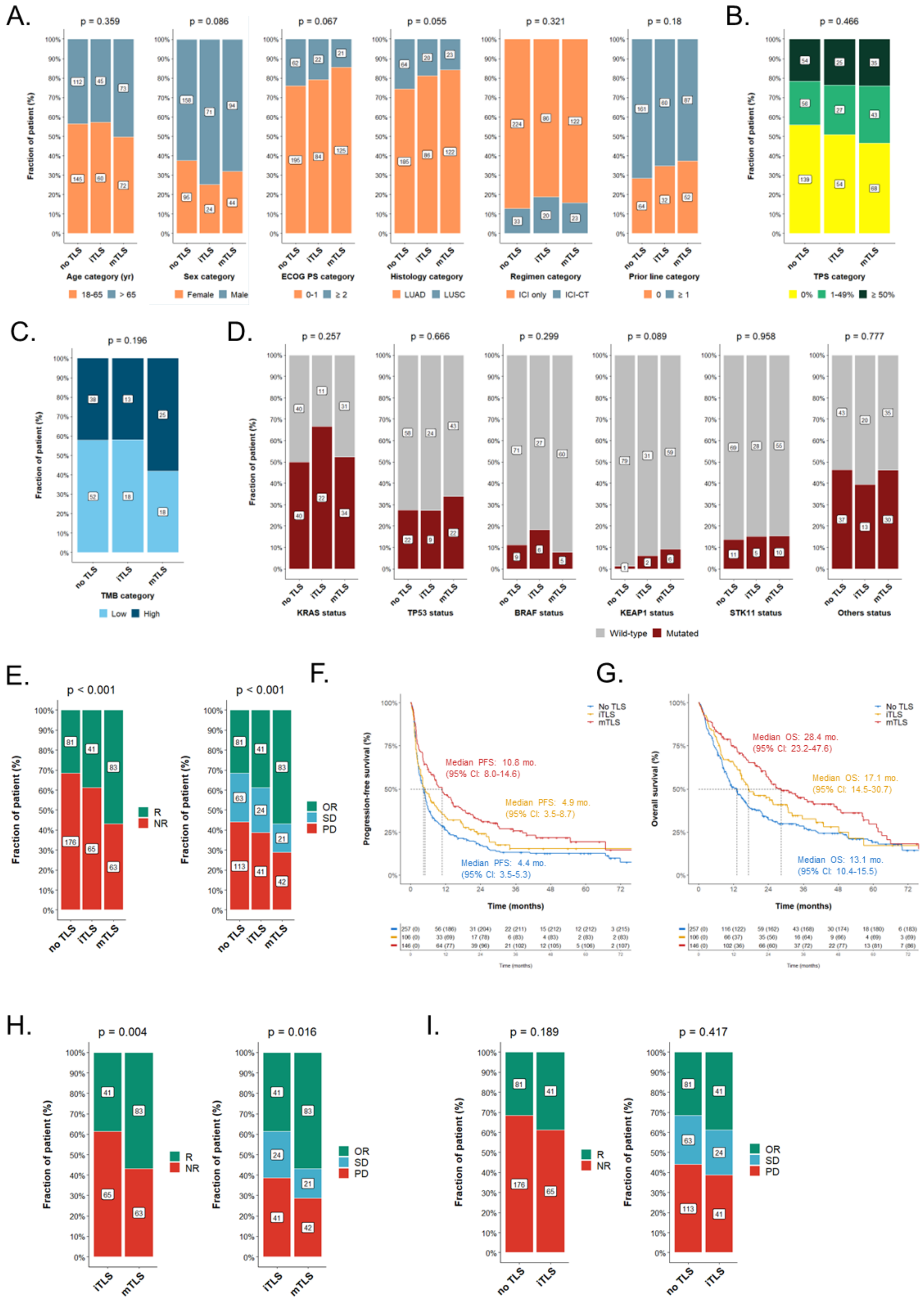

Figure S1. Characterization of ICI-treated NSCLC patients based on TLS status. Related to Figure 1.

A. Distribution of baseline characteristics of patients according to the TLS status. Statistical significance was determined by chi-squared test.

B. Distribution of PD-L1 tumor expression (TPS) of NSCLC samples according to the TLS status. Statistical significance was determined by chi-squared test.

C. Distribution of tumor mutational burden (TMB) of NSCLC samples according to the TLS status. Statistical significance was determined by chi-squared test.

D. Distribution of mutational status of NSCLC samples according to the TLS status. Statistical significance was determined by chi-squared test.

E. Response rate, as defined per objective response (left) or RECIST 1.1 criteria (right), according to TLS status: absence (negative, no TLS) , iTLS or mTLS. Statistical significance was determined by chi-squared test.

F. Kaplan–Meier analysis of the PFS of patients according to TLS status (n=509; red curve: mature TLS-enriched tumors; orange curve: immature TLS-enriched tumors; blue curve: TLS-negative tumors). Numbers below each x axis indicate the number of patients at risk and those in parentheses are the number of events.

G. Kaplan–Meier analysis of the OS of patients according to TLS status (n=509; red curve: mature TLS-enriched tumors; orange curve: immature TLS-enriched tumors; blue curve: TLS-negative tumors). Numbers below each x axis indicate the number of patients at risk and those in parentheses are the number of events.

H. Response rate, as defined per objective response (left) or RECIST 1.1 criteria (right), according to TLS status: immature TLS (iTLS) or mature TLS (mTLS). Statistical significance was determined by chi-squared test.

I. Response rate, as defined per objective response (left) or RECIST 1.1 criteria (right), according to TLS status: absence (no TLS) or immature TLS (iTLS). Statistical significance was determined by chi-squared test.

CI: confidence interval; ECOG PS: Eastern Cooperative Oncology Group Performance Status; ICI: immune checkpoint inhibitors; iTLS: immature TLS; mTLS: mature TLS; no TLS: absence of TLS; OR, objective response; PD, progressive disease; SD, stable disease; R, responder; NR, non-responder.

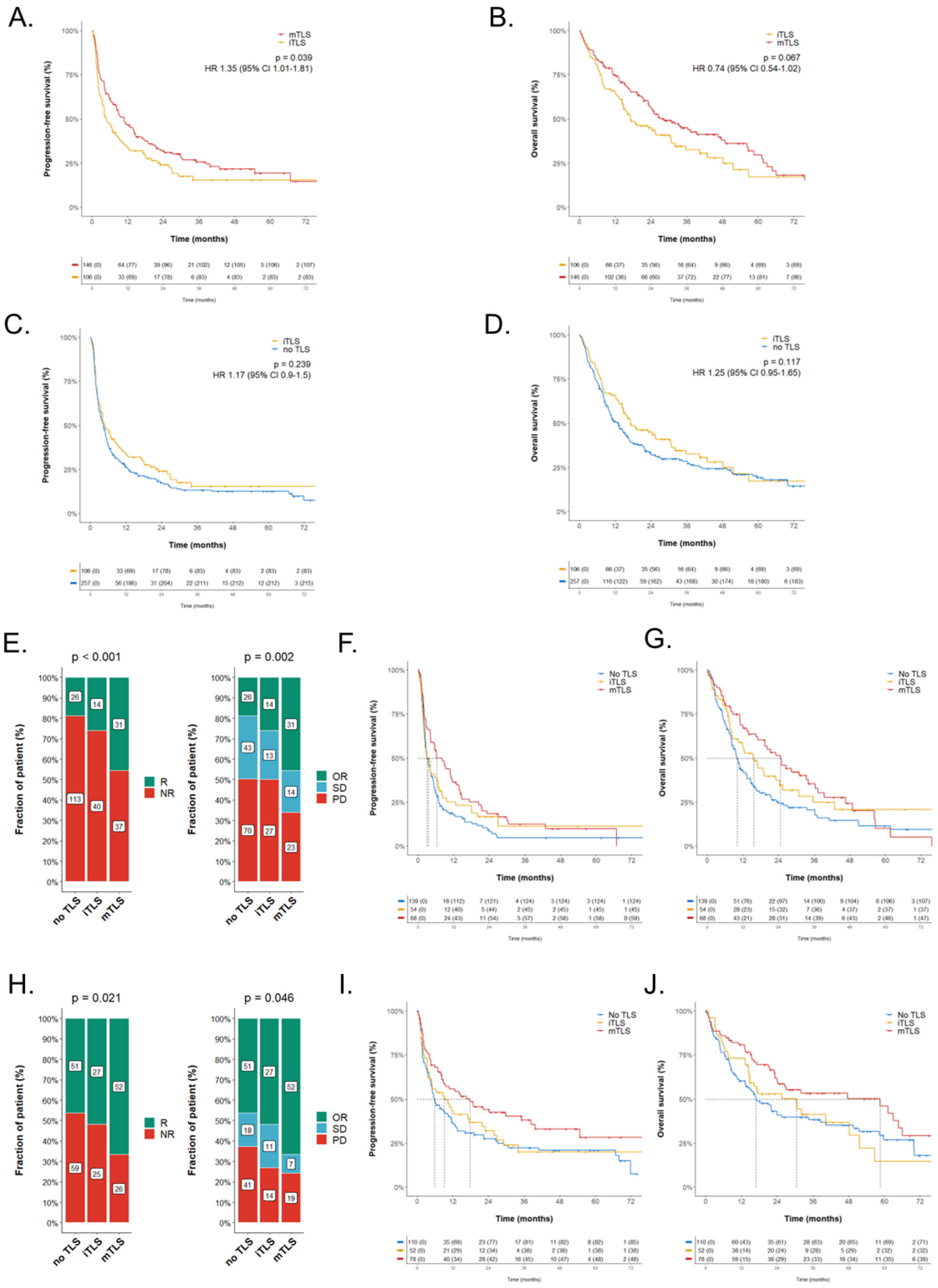

Figure S2. Clinical outcomes of ICI-treated NSCLC patients based on TLS status. Related to Figure 1.

A. Kaplan–Meier curves of the PFS of patients according to TLS maturity status (red curve: mTLS-enriched tumors; orange curve: iTLS-enriched tumors). Numbers below each x axis indicate the number of patients at risk and those in parentheses are the number of events. Statistical significance was determined by log-rank test.

B. Kaplan–Meier curves of the OS of patients according to TLS maturity status (red curve: mTLS-enriched tumors; orange curve: iTLS-enriched tumors). Numbers below each x axis indicate the number of patients at risk and those in parentheses are the number of events. Statistical significance was determined by log-rank test.

C. Kaplan–Meier curves of the PFS of patients according to TLS maturity status (blue curve: TLS-negative tumors; orange curve: iTLS-enriched tumors). Numbers below each x axis indicate the number of patients at risk and those in parentheses are the number of events. Statistical significance was determined by log-rank test.

D. Kaplan–Meier curves of the OS of patients according to TLS maturity status (blue curve: TLS-negative tumors; orange curve: iTLS-enriched tumors). Numbers below each x axis indicate the number of patients at risk and those in parentheses are the number of events. Statistical significance was determined by log-rank test.

E. Response rate, as defined per objective response (left) or RECIST 1.1 criteria (right), according to TLS status in PD-L1 negative tumors. Statistical significance was determined by chi-squared test.

F. Kaplan–Meier curves of the PFS of patients according to TLS maturity status in PD-L1 negative tumors (red curve: mTLS-enriched tumors; orange curve: iTLS-enriched tumors; blue curve: TLS-negative tumors). Numbers below each x axis indicate the number of patients at risk and those in parentheses are the number of events.

G. Kaplan–Meier curves of the OS of patients according to TLS maturity status in PD-L1 negative tumors (red curve: mTLS-enriched tumors; orange curve: iTLS-enriched tumors; blue curve: TLS-negative tumors). Numbers below each x axis indicate the number of patients at risk and those in parentheses are the number of events.

H. Response rate, as defined per objective response (left) or RECIST 1.1 criteria (right), according to TLS status in PD-L1 positive tumors. Statistical significance was determined by chi-squared test.

I. Kaplan–Meier curves of the PFS of patients according to TLS maturity status in PD-L1 positive tumors (red curve: mTLS-enriched tumors; orange curve: iTLS-enriched tumors; blue curve: TLS-negative tumors). Numbers below each x axis indicate the number of patients at risk and those in parentheses are the number of events.

J. Kaplan–Meier curves of the OS of patients according to TLS maturity status in PD-L1 positive tumors (red curve: mTLS-enriched tumors; orange curve: iTLS-enriched tumors; blue curve:

TLS-negative tumors). Numbers below each x axis indicate the number of patients at risk and those in parentheses are the number of events.

ECOG PS: Eastern Cooperative Oncology Group Performance Status; ICI: immune checkpoint inhibitors; iTLS: immature TLS; mTLS: mature TLS; no TLS: absence of TLS; OR, objective response; PD, progressive disease; SD, stable disease; R, responder; NR, non-responder.

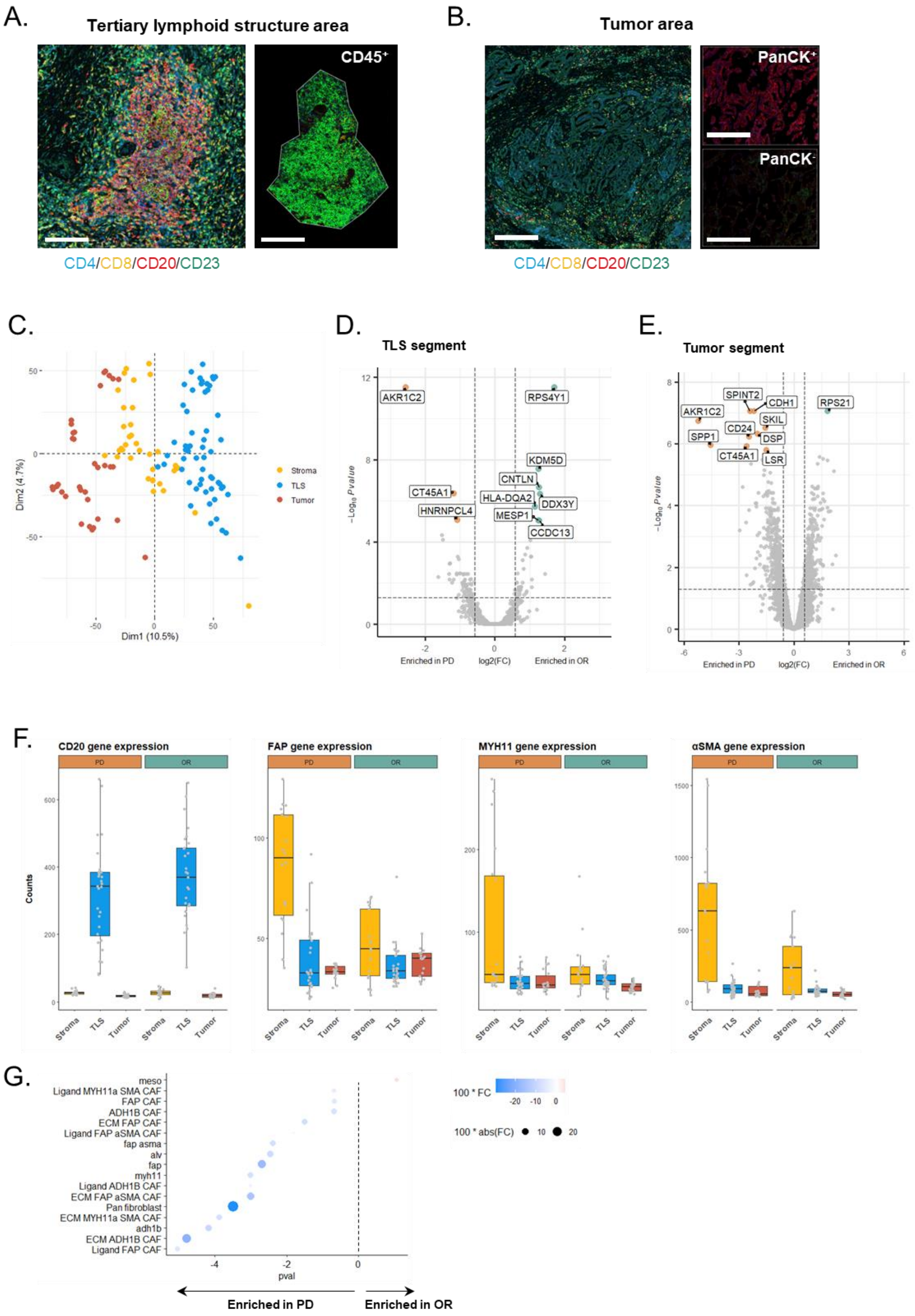

Figure S3. Spatial transcriptomic analysis of ICI-treated TLS-positive NSCLC. Related to Figure 2.

A. Representative illustration of a GeoMx DSP-analyzed TLS area. CD4/CD8/CD20/CD23 multiplex IHF staining (left) and CD45 (right) segmentation mask for GeoMx transcriptomics analysis. All scale bars, 50µm.

B. Representative illustration of a GeoMx DSP-analyzed tumor area. CD4/CD8/CD20/CD23 multiplex IHF staining (left) and PanCK (right) segmentation masks (righ panel, positive cells [top] and negative cells [bottom]) for GeoMx transcriptomics analysis. All scale bars, 200µm.

C. Principal component analysis (PCA) plot displaying AOIs according to segment.

D. Volcano plot of differentially genes expression between responders (PD, N=3) and non responders (OR, N=3) in TLS segment. Top 10 upregulated genes are displayed.

E. Volcano plot of differentially genes expression between responders (PD, N=3) and non responders (OR, N=3) in tumor segment. Top 10 upregulated genes are displayed.

F. Gene expression of CD20, FAP, MYH11 and aSMA in TLS, tumor and stroma segments, respectively, between non responders (PD, N=3) and responders (OR, N=3). Data are represented as median +/- IQR.

G. Bubble plot of publicly available scRNAseq gene signature from Grout et al. (TCGA and external dataset) estimated with deconvolution algorithm between non responders (PD, N=3) and responders (OR, N=3) or the GeoMx spatial transcriptomic experiment.

FC: fold change; IQR: interquartile range; TLS: tertiary lymphoid structure.

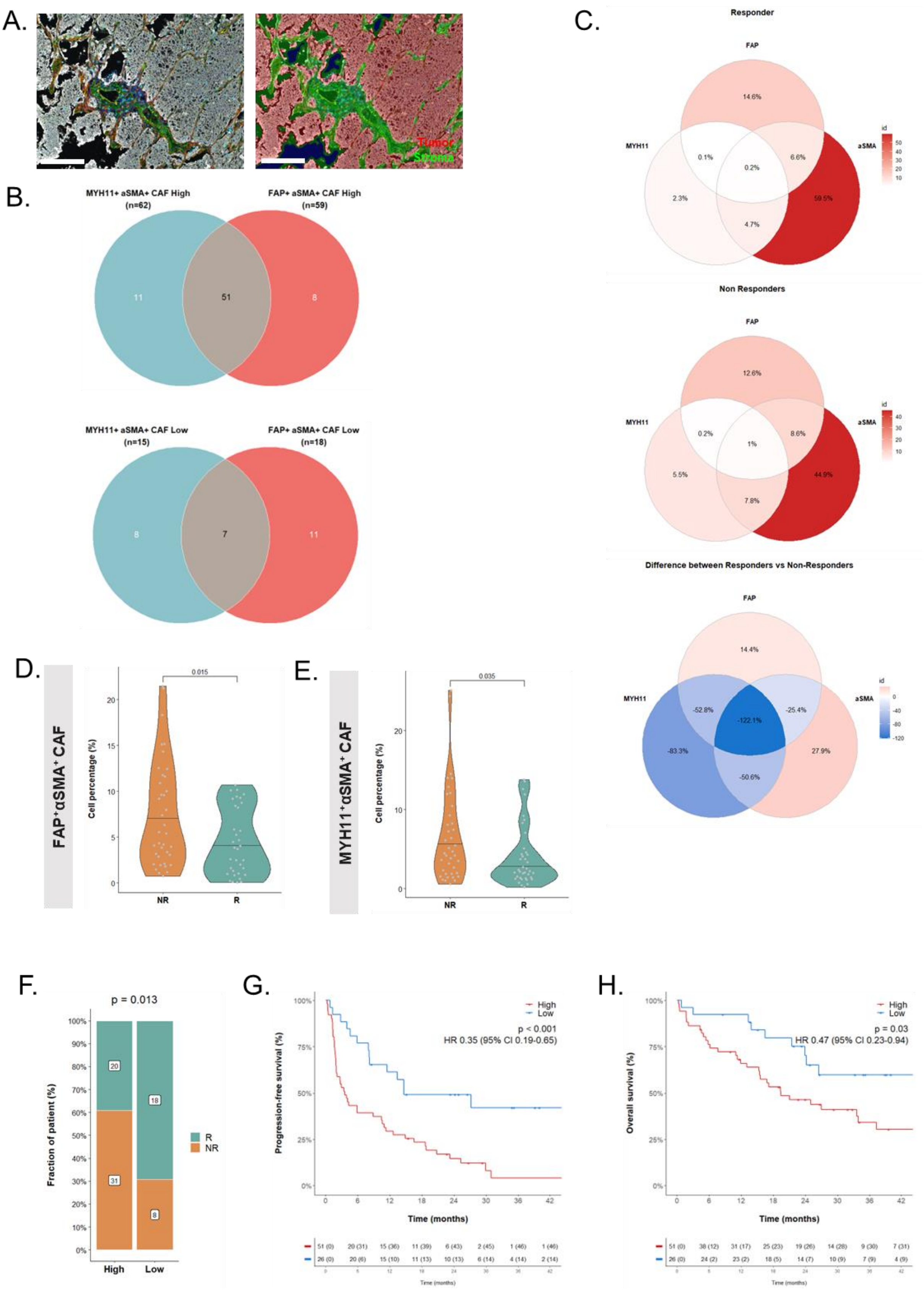

Figure S4. Clinical outcomes of ICI-treated TLS-positive NSCLC patients based on CAFs status. Related to Figure 3.

A. Illustration of the segmentation strategy of the tissue in “Stroma” and “Tumor” areas. All scale bars, 200µm.

B. Venn diagram depicting individuals CAF category with MYH11$^+$αSMA$^+$ CAF and/or FAP$^+$αSMA$^+$ CAF High (upper) and with MYH11$^+$αSMA$^+$ CAF and/or FAP$^+$αSMA$^+$ CAF Low (bottom).

C. Venn diagram of CAF marker expression of NSCLC Responders (upper - R) and Non-Responders (middle - NR). Differences in cells proportion between responders and non-responders are displayed on bottom. For the analysis, cohort (n=77 patients) was down-sampled to 5000 cells per patient. Median of cell distribution is shown.

D. Percentage of FAP$^+$αSMA$^+$ CAF in the stroma areas of responders and non-responders to ICI. The P values were calculated using Wilcoxon tests. Data are represented as median.

E. Percentage of MYH11$^+$αSMA$^+$ CAF in the stroma areas of responders and non-responders to ICI. The P values were calculated using Wilcoxon tests. Data are represented as median.

F. Proportion of patients with high and low density of combined FAP$^+$αSMA$^+$ and MYH11$^+$αSMA$^+$ CAF according to response. The P value was calculated using a X2 test.

G. Kaplan–Meier curves of the PFS of patients classified as high or low based on levels of combined FAP$^+$αSMA$^+$ and MYH11$^+$αSMA$^+$ CAF .

H. Kaplan–Meier curves of the OS of patients classified as high or low based on levels of combined FAP$^+$αSMA$^+$ and MYH11$^+$αSMA$^+$ CAF .

CAF: cancer-associated fibroblast; ICI: immune checkpoint inhibitor; NSCLC: non-small cell lung cancer.

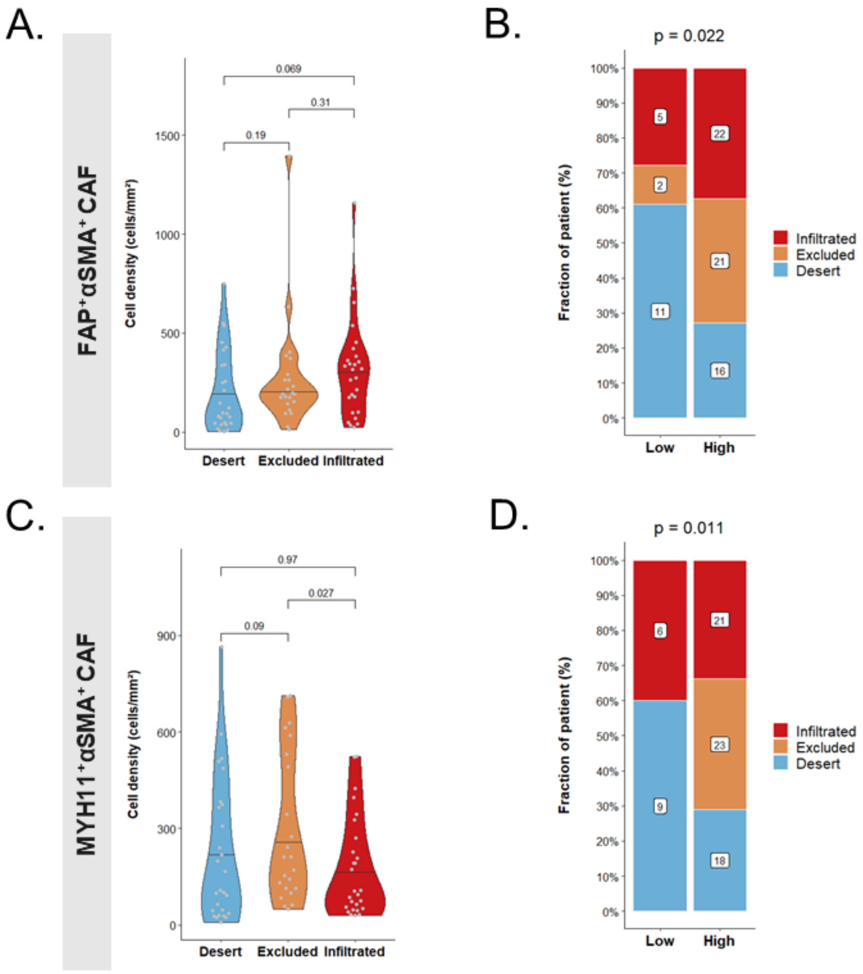

Figure S5. Analysis of CAF density and category based on immune contexture. Related to Figure 3.

A. Density of FAP$^+$αSMA$^+$ CAF in the stroma according to immune contexture. The P values were calculated using Wilcoxon tests. Data are represented as median.
B. Proportion of patients with infiltrated, excluded and desert immune contexture according to density of FAP$^+$αSMA$^+$ CAF category. The P value was calculated using chi-squared test.
C. Density of MYH11$^+$αSMA$^+$ CAF in the stroma according to immune contexture. The P values were calculated using Wilcoxon tests. Data are represented as median.
D. Proportion of patients with infiltrated, excluded and desert immune contexture according to density of MYH11$^+$αSMA$^+$ CAF category. The P value was calculated using chi-squared test.

CAF: cancer-associated fibroblast.

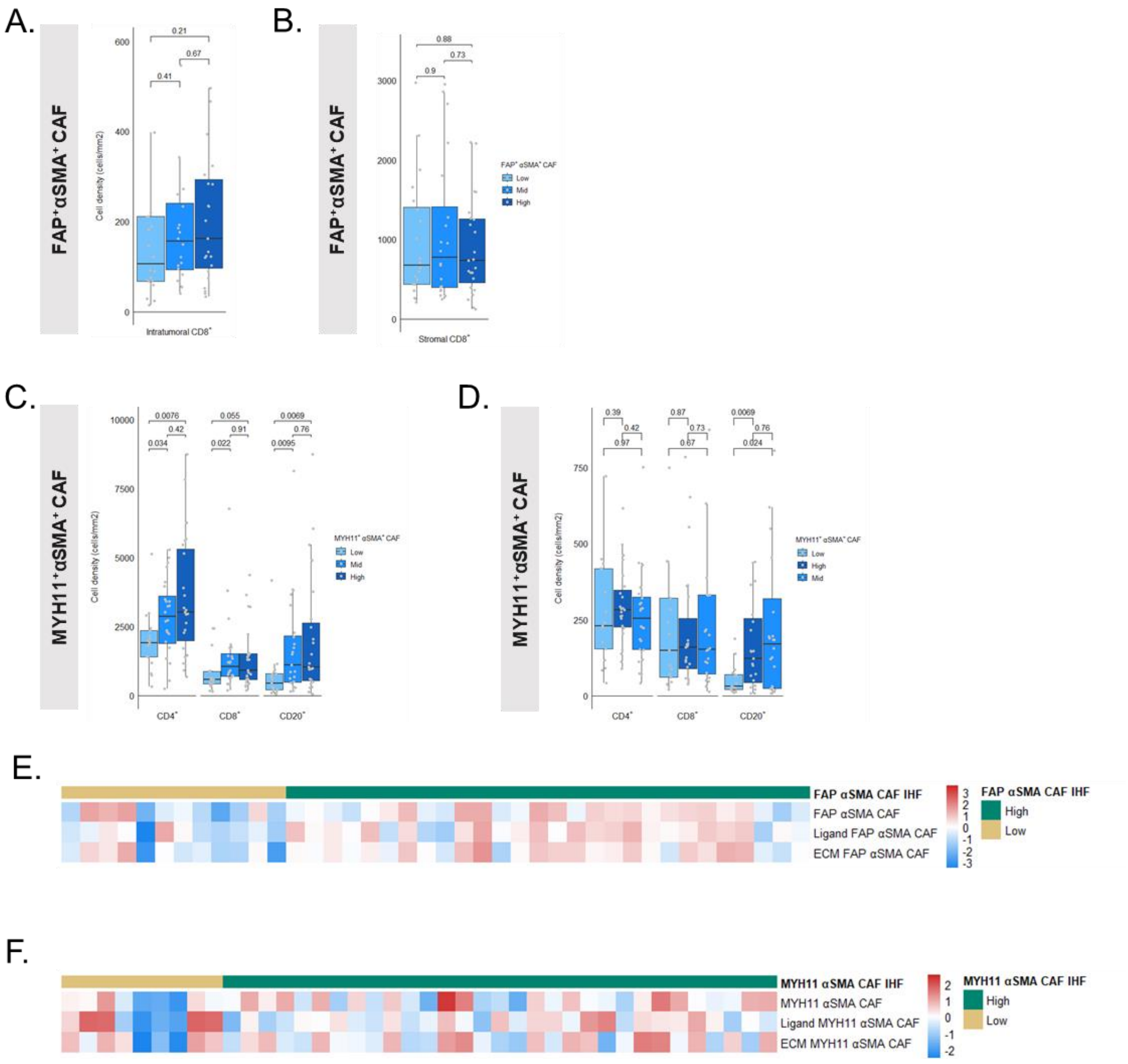

Figure S6. Analysis of T and B cells infiltrate based on CAF subsets. Related to Figure 4 and 5.

A. Density of intratumoral CD8$^+$ T cells according to stromal FAP$^+$αSMA$^+$ CAF category. The P values were calculated using Wilcoxon tests. Data are represented as median +/- IQR.

B. Density of stromal CD8$^+$ T cells according to stromal FAP$^+$αSMA$^+$ CAF category. The P values were calculated using Wilcoxon tests. Data are represented as median +/- IQR.

C. Density of stromal CD4$^+$, CD8$^+$ and CD20$^+$ T cells according to stromal MYH11$^+$αSMA$^+$ CAF category. The P values were calculated using Wilcoxon tests. Data are represented as median +/- IQR.

D. Density of intratumoral CD4$^+$, CD8$^+$ and CD20$^+$ T cells according to stromal MYH11$^+$αSMA$^+$ CAF category. The P values were calculated using Wilcoxon tests. Data are represented as median +/- IQR.

E. Heatmap of scRNAseq FAP^{+}αSMA^{+} CAF, its ligand or its ECM signatures, respectively, according to FAP^{+}αSMA^{+} CAF IHF category. FAP^{+}αSMA^{+} CAF phenotype is identified by IHF on the matched FFPE samples and then used to stratify samples.

F. Heatmap of scRNAseq MYH11^{+}αSMA^{+} CAF, its ligand or its ECM signatures, respectively, according to MYH11^{+}αSMA^{+} CAF IHF category. MYH11^{+}αSMA^{+} CAF phenotype is identified by IHF on the matched FFPE samples and then used to stratify samples.

CAF: cancer-associated fibroblasts; IQR: interquartile range.

Clinical characteristic		N (%)
Age - yr	Median (range)	63.8 (30.0 - 92.0)
Age group	18-65	278 (54.6)
	>65	231 (45.4)
Sex	Female	168 (33.0)
	Male	341 (67.0)
Histotype	Adenocarcinoma	393 (77.2)
	Squamous cell carcinoma	107 (21.0)
	Others*	9 (1.8)
Sample type	Needle biopsy	410 (80.6)
	Surgical resection	99 (19.4)
Performance status	0-1	404 (79.4)
	≥ 2	105 (20.6)
Prior line of treatment	0	148 (29.1)
	≥ 1	308 (60.5)
	NA	53 (20.4)
Regimen	ICI only	432 (84.9)
	ICI-CT combination	77 (15.1)
TPS	Negative	261 (51.2)
	1-49%	126 (24.8)
	≥ 50%	114 (22.4)
	NE	8 (1.6)
* Others: adenosquamous, neuroendocrine, sarcomatoid and undifferenciated carcinoma.		
Abbreviations: CT: chemotherapy; ICI: immune checkpoint inhibitors; NA: not available; NE: not evaluable; TPS: tumor proportion score.		

Table S1. Baseline characteristics of NSCLC patients (N=509). Related to Figure 1, Figure S1, Figure S2 and Table 1.

	OR group			PD group		
Clinical characteristic	P1	P2	P3	P4	P5	P6
Age	63	70	74	69	69	56
Sex	Male	Male	Male	Male	Male	Female
Histotype	Adenocarcinoma	Adenocarcinoma	Adenocarcinoma	Adenocarcinoma	Adenocarcinoma	Adenocarcinoma
Performance status	0	0	1	0	1	1
Prior line	1	5	2	2	1	2
Sample site	Lung	Adrenal	Lung	Lung	Lung	Lung
TPS (PD-L1, %)	10	0	0	0	95	0
Molecule	Nivolumab	Nivolumab	Nivolumab	Nivolumab	Pembrolizumab	Nivolumab
PFS	50.0	10.8	23.3	1.1	2	1.3
OS	86.8	36.4	37.3	4.7	11.7	10.0
Abbreviations: OR: objective response; OS; overall survival; PD: progressive disease; PFS: progression-free survival; TPS: tumor proportion score.						

Table S2. Clinical characteristics of patients with mTLS-positive NSCLC of the spatial transcriptomic experiment. Related to Figure 2, Figure S3, Table S3 and Table S4.

		ROI			Nuclei per AOI		
		TLS	Tumor	Stroma	TLS	Tumor	Stroma
OR group	P1	10	6	6	10614	9505	6083
	P2	10	6	6	19207	10784	9849
	P3	10	6	6	15439	10649	11252
PD group	P4	9	6	6	5828	9147	11810
	P5	10	6	6	8129	11035	8721
	P6	10	6	6	10381	12121	7707
Abbreviations: AOI: area of interest; mTLS: mature tertiary lymphoid structure; OR: objective response; PD: progressive disease; ROI: region of interest.							

Table S3. Number of ROI and nuclei per AOI for each patients with TLS-positive NSCLC of the spatial transcriptomic experiment. Related to Figure 2, Figure S3, Table S2 and Table S4.

Reference	https://doi.org/10.3390/cancers14051290	https://doi.org/10.1038/s41467-019-12464-3	https://doi.org/10.1038/s42003-022-04356-y	https://doi.org/10.1172/jci128672	https://doi.org/10.1016/j.immuni.2016.10.021	https://doi.org/10.1186/s13059-015-0620-6
Name	Schroeder 2022	Szabo 2019	Devi-Marulkar 2023	Freeman 2020	DeSimone 2016	Angelova 2015
List of genes	CD2	PMCH	FOXP3	ICOS	ADORA2A	CCL19
	CD247	FOXP3	TNFRSF9	CTLA4	TNFRSF18	CD34
	CD27	TIGIT	BTLA	TIGIT	LAG3	CD72
	CD28	CTLA4	TNFRSF4	CD28	LGALS9	CTLA4
	CD3D	IL2RA	PDCD1	TNFSF8	TNFRSF8	FOXP3
	CD3E	TNFRSF4	CD276	CD27	CD44	GADD45B
	CD3G	TNFRSF18	LAG3	CD226	TNFRSF4	GEM
	CD4	SELL	CCL20	TNFRSF9	PDCD1	IL1RL1
	CD5	ID3	CCL22	CD274	TNFRSF9	IL9R
	CD6	IFIT3	CXCL5	HAVCR2	ICOS	MADCAM1
	CD70	IFI6	CXCR3	CEACAM1	CTLA4	MYH10
	CD96	MAF	TNF	ENTPD1	TNFRSF14	NCF2
	CTLA4	STAT1	STAT4	LAG3	LAIR1	RCSD1
	DPP4	MAL	FOXA1	TNFRSF4	HAVCR2	RYR1
	FOXP3	LGALS3	IL10	TNFRSF18	TNFSF14	SELE
	ICOS	CCR7	ENTPD1	CD80	CD27	SELP
	IL2RA	PDZD8	LRRC32	BTLA	CD86	SFRP1
	IL2RB	CXCR6		CD40LG	CD70	SIT1
	LCK	AQP3			CD200R1	TIGIT
	LTB	SOCS1			TNFSF4	TLR10
	SH2D1A	MIR155HG			C10orf54	TLR2
	TRAT1	ANXA2			TIGIT	TLR7
	ZAP70	PSAT1			CD80	TLR8
		WARS			TNFRSF25	TRAF1
					CD40LG	WIPF1
					CD28	TGFB1
					IDO2	
					CD244	
					TMIGD2	

Table S7. RNAseq signatures of regulatory T cells pathway. Related to Figure 5 and Table S8.
