# Supplementary material for: Spatially resolved transcriptomics reveal the determinants of primary resistance to immunotherapy in NSCLC with mature tertiary lymphoid structures
Source: Cell Rep Med. 2025 Feb 4;6(2):101934. doi: 10.1016/j.xcrm.2025.101934 (PMC11866545; doi:10.1016/j.xcrm.2025.101934)
Supplement: Document S2. Article plus supplemental information [file mmc6.pdf]

Cell Reports
Medicine

Article

Spatially resolved transcriptomics reveal the determinants of primary resistance to immunotherapy in NSCLC with mature tertiary lymphoid structures

Graphical abstract

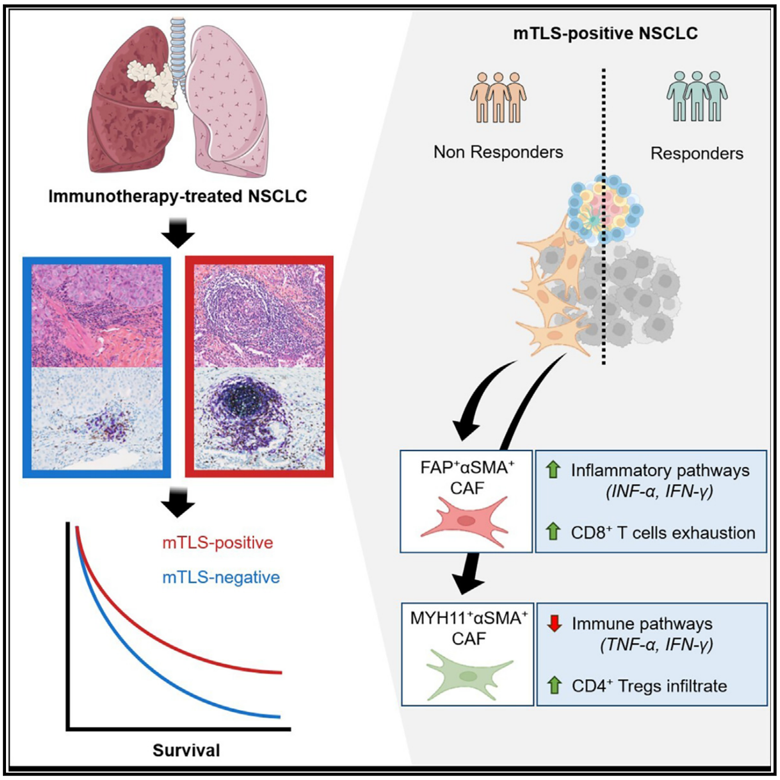

Highlights

- mTLSs are predictive of response to ICIs in NSCLC
- Two CAF subsets within the TME are key determinants of primary resistance to ICIs
- $FAP^{+}\alpha SMA^{+}$ CAFs correlate with the inflammatory response and exhaustion of $CD8^{+}$ T cells
- $MYH11^{+}\alpha SMA^{+}$ CAFs favor an immunosuppressive TME with $CD4^{+}$ Treg cell infiltration

Authors

Florent Peyraud, Jean-Philippe Guégan, Christophe Rey, ..., Robert J. Johnston, Alban Bessede, Antoine Italiano

Correspondence

a.italiano@bordeaux.unicancer.fr

In brief

Peyraud et al. show that the presence of mTLSs is associated with improved clinical outcome with immunotherapy in NSCLC. However, primary resistance is driven by two distinct subsets of cancer-associated fibroblasts that are associated with immune exclusion, $CD8^{+}$ T cell exhaustion, and increased regulatory $CD4^{+}$ T cell infiltration.

Peyraud et al., 2025, Cell Reports Medicine 6, 101934
February 18, 2025 © 2025 The Author(s). Published by Elsevier Inc.
https://doi.org/10.1016/j.xcrm.2025.101934

CellPress

Cell Reports Medicine

CellPress
OPEN ACCESS

Article

Spatially resolved transcriptomics reveal the determinants of primary resistance to immunotherapy in NSCLC with mature tertiary lymphoid structures

Florent Peyraud,[1,2,3] Jean-Philippe Guégan,[3] Christophe Rey,[3] Oren Lara,[3] Ophélie Odin,[3] Marie Del Castillo,[4] Lucile Vanhersecke,[5] Jean-Michel Coindre,[2,5] Emma Clot,[6] Maxime Brunet,[1] Thomas Grellety,[6] Angélique Tasseel,[7] Sylvestre Le Moulec,[7] Robert J. Johnston,[8,9] Alban Bessede,[3] and Antoine Italiano[1,2,9,10,*]
[1]Department of Medicine, Institut Bergonié, Bordeaux, France
[2]Faculty of Medicine, University of Bordeaux, Bordeaux, France
[3]Explicyte Immuno-Oncology, Bordeaux, France
[4]Atlantic Pathologie, Saint-Pierre-d'Irube, France
[5]Department of Pathology, Institut Bergonié, Bordeaux, France
[6]Centre Hospitalier de la Côte Basque, Bayonne, France
[7]Clinique Marzet, Pau, France
[8]Department of Cancer Immunology, Genentech, A member of the imCORE Network, South San Francisco, CA, USA
[9]DITEP, Gustave Roussy, Villejuif, France
[10]Lead contact
*Correspondence: a.italiano@bordeaux.unicancer.fr
https://doi.org/10.1016/j.xcrm.2025.101934

SUMMARY

Effectiveness of immune checkpoint inhibitors (ICIs) in non-small cell lung cancer (NSCLC) has been linked to the presence of mature tertiary lymphoid structures (mTLSs) within the tumor microenvironment (TME). However, only a subset of mTLS-positive NSCLC derives benefit, thus highlighting the need to unravel ICI response determinants. The comprehensive analysis of ICI-treated patients with NSCLC (*n* = 509) from the Bergonié Institute Profiling (BIP) study (NCT02534649) reveals that the presence of mTLSs correlates with improved clinical outcomes, independently of programmed death ligand 1 (PD-L1) expression and genomic features. Employing spatial transcriptomics alongside multiplex immunofluorescence (mIF), we show that two distinct subsets of cancer-associated fibroblasts (CAFs) are essential factors in mediating primary resistance to ICIs in mTLS-positive NSCLC. These CAFs are associated with immune exclusion, $CD8^+$ T cell exhaustion, and increased regulatory $CD4^+$ T cell infiltration, underscoring an immunosuppressive TME. Our study highlights the pivotal role of specific CAF subsets in thwarting ICIs, proposing new therapeutic targets to enhance immunotherapy efficacy.

INTRODUCTION

Non-small cell lung cancer (NSCLC) represents a major hurdle in oncology, characterized by its aggressive nature and dismal prognosis, particularly in its advanced or metastatic stages.[1] The emergence of immunotherapy has revolutionized the treatment landscape of NSCLC, offering newfound hope to patients.[2] Monoclonal antibodies targeting programmed death 1 (PD1) and its ligand, PD-L1, have demonstrated significant efficacy, either as monotherapy for individuals with high PD-L1 expression or in combination with platinum-based chemotherapy, leading to improvement in survival.[3–8] Despite these substantial strides, resistance to immune checkpoint inhibitors (ICIs) remains a significant obstacle, with the underlying mechanisms poorly elucidated.[9–11]

Tertiary lymphoid structures (TLSs) within the tumor microenvironment (TME) have emerged as critical regulators of the anti-tumor immune response.[12,13] Previous studies, including our own, have highlighted the favorable impact of mature TLS (mTLS) presence on treatment outcomes in patients with advanced cancer receiving anti-PD1/PD-L1 therapy. In this retrospective analysis evaluating over 500 patients with solid tumors treated with anti-PD1/PD-L1 monoclonal antibodies, mTLS presence correlated significantly with enhanced response rates, prolonged progression-free survival (PFS), and improved overall survival (OS).[14] Notably, this predictive value was specific to immunotherapy, as mTLS presence did not impact outcomes in patients treated with chemotherapy alone, suggesting its intrinsic predictive rather than prognostic value.[15]

Validation of TLS predictive utility was further underscored in large randomized trials. Through comprehensive analysis, Patil et al. investigated the predictive significance of a B cell transcriptional signature in patients with NSCLC treated with

This is an open access article under the CC BY license (http://creativecommons.org/licenses/by/4.0/).

Table 1. Baseline characteristics of patients with mTLS-positive versus mTLS-negative NSCLC (*N* = 509)

Clinical characteristic		Presence of mTLS (*n* = 146)	Absence of mTLS (*n* = 363)
Age, year	median (range)	63.7 (34.8–92.0)	63.8 (30.0–86.2)
Age group	18–65	72 (49.3)	205 (56.5)
	>65	74 (50.7)	158 (43.5)
Sex	female	44 (30.1)	124 (34.2)
	male	102 (69.9)	239 (65.8)
Histotype	adenocarcinoma	122 (83.6)	271 (74.7)
	squamous cell carcinoma	23 (15.8)	84 (23.1)
	others[a]	1 (0.7)	9 (2.2)
Sample type	needle biopsy	82 (56.2)	328 (90.4)
	surgical resection	64 (43.8)	35 (9.6)
Performance status	0–1	125 (85.6)	279 (76.9)
	≥2	21 (14.4)	84 (23.1)
Prior line of treatment	0	52 (35.6)	96 (26.4)
	≥1	87 (59.6)	222 (61.2)
	NA	7 (4.8)	45 (12.4)
Regimen	ICI only	122 (83.6)	310 (85.4)
	ICI-CT combination	24 (16.4)	53 (14.6)
TPS	Negative	68 (46.6)	193 (53.2)
	1%–49%	43 (29.4)	83 (22.9)
	≥50%	35 (24.0)	79 (21.8)
	NE	0 (0.0)	8 (2.1)

Abbreviations: CT, chemotherapy; ICI, immune checkpoint inhibitor; NA, not available; NE, not evaluable; NSCLC, non-small cell lung cancer; mTLS, mature tertiary lymphoid structure; TPS, tumor proportion score.
[a]Others: adenosquamous, neuroendocrine, sarcomatoid, and undifferentiated carcinoma.

either anti-PD-L1 atezolizumab or docetaxel-based chemotherapy. Their findings revealed a compelling association between extended survival outcomes, specifically with atezolizumab, and a B cell expression signature.[16] The presence of TLSs and organized lymphoid aggregates was further confirmed in a few subsets of patients with increased B cell-derived plasma cell signatures.[16] Importantly, this predictive capacity remained robust even after accounting for conventional predictive biomarkers such as CD8$^+$ T cell density, PD-L1 expression, and tumor mutational burden. Notably, TLS presence failed to confer predictive benefit for patients treated with chemotherapy-based regimen docetaxel, underscoring the specificity of TLSs as a specific predictive biomarker for immunotherapy response.[16] These insights highlight the differential predictive value of TLSs across treatment modalities, further accentuating their potential as a prognostic tool specifically tailored for immunotherapeutic interventions.

These collective findings delineate a distinct subset of NSCLC characterized by the presence of TLSs, exhibiting unique biological features and heightened sensitivity to immunotherapy. Nonetheless, a noteworthy proportion of patients with TLS-positive NSCLC fail to derive benefit from immune checkpoint inhibition. Thus, the primary objectives of our study were to validate, in the largest cohort of patients with lung cancer ever explored, the predictive value of the histological assessment of mTLSs and to elucidate the determinants of resistance to ICIs within this specific biological entity. Identification of mechanisms of resistance to immunotherapy in mTLS-positive NSCLC may help to define new therapeutic strategies to enhance immunotherapy efficacy in this group of patients.

RESULTS

mTLSs are associated with clinical outcomes to ICIs in NSCLC independently of PD-L1 and genomic features

In a comprehensive analysis of 509 patients with locally advanced or metastatic NSCLC, we sought to elucidate the impact of TLS status and maturity on clinical outcomes of patients treated with ICI-based regimen. Baseline characteristics of patients are summarized in Table 1 (Table S1). TLS status, evaluated by trained pathologists on pre-treatment tumor samples, was found in 49.5% (252/509) of cases, with 20.8% characterized as immature TLSs (iTLSs) and 28.7% as mTLSs, identified by the presence of CD23$^+$ follicular dendritic cells (Figure 1A). Baseline characteristics did not significantly differ with TLS status (Figure S1A), and their presence was noted across various PD-L1 tumor expression categories (Figure S1B). Genomic profiling revealed no significant association between TLS status and tumor mutational profiles (Figures 1B, S1C, and S1D).

We found that the presence of mTLSs was significantly higher in patients who were long-term survivors, achieving an OS of 24 months or more. Specifically, 41.3% of these long-term

CellPress
OPEN ACCESS

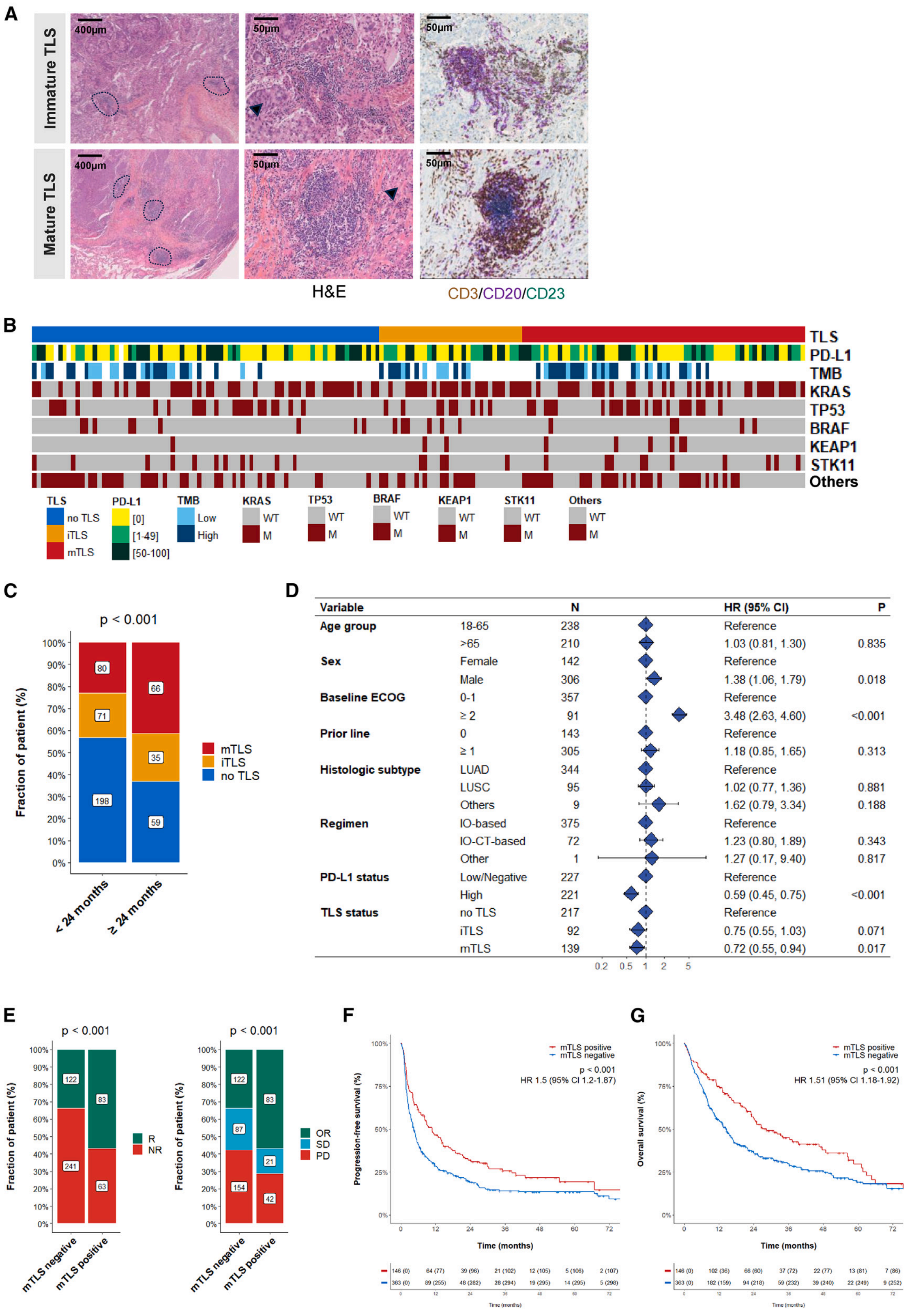

(legend on next page)

survivors had mTLS-positive tumors, a significantly higher proportion than the 22.9% observed in other patients (66/160, 95% confidence interval [CI]: 33.6–49.3 versus 80/349, 95% CI: 18.7–27.8, respectively; $p < 0.001$; Figure 1C). Baseline Eastern Cooperative Oncology Group (ECOG) performance status and positive PD-L1 tumor expression, along with TLS maturity, were univariately associated with improved OS. In multivariate analysis, OS remained significantly correlated with mTLSs, independently of PD-L1 expression and other covariates (Figure 1D).

Response rates to anti-PD1/PD-L1 antibody-based treatment were 56.8% in the mTLS-positive cohort, notably higher than the 33.6% in the mTLS-negative group (83/146, 95% CI: 48.4–64.9 versus 122/363, 95% CI: 28.8–38.8, respectively; $p < 0.001$; Figure 1E). This enhanced response was consistent across objective response rates by response evaluation criteria in solid tumors (RECIST) criteria (Figure 1E). Similarly, the proportion of responders remained significantly higher in patients with mTLS-positive tumors compared to those with iTLSs (41/106, 38.7%, $p = 0.004$; Figure S1H). Notably, no substantial differences in response rates were observed between the no TLS and iTLS subgroups (Figure S1I).

At a median follow-up of 28.7 months, the median PFS favored mTLS-positive patients, at 10.8 months (95% CI: 8.0–14.6) compared to 4.5 months (95% CI: 3.7–5.3) in the mTLS-negative group ($p < 0.001$; Figure 1F). Similarly, the median OS was more favorable in the mTLS-positive cohort, with 28.4 months (95% CI: 23.2–47.6) compared to 14.5 months (95% CI: 12.1–16.7) for mTLS-negative patients ($p < 0.001$; Figure 1G). Both 6-month, 1-year, and 2-year PFS and OS rates were notably higher in the mTLS-positive group. Notably, survival differences favored the mTLS-positive subgroup in comparison with the iTLS-positive subgroup (Figures S2A and S2B). Importantly, no differences in survival were detected between the iTLS and no TLS subgroups (Figures S2C and S2D). Our findings demonstrate that the presence of mTLS predicts a better outcome in patients with NSCLC treated with immunotherapy, regardless of PD-L1 expression (Figures S2E–S2J), underscoring mTLS status as a robust independent biomarker for patient stratification and prognosis.

The presence of fibroblasts negatively impacts outcomes to ICIs in mTLS-positive NSCLC

To elucidate the determinants of ICI response in mTLS-positive NSCLC, we conducted a comprehensive spatial profiling of gene expression using the GeoMx Whole Transcriptome Atlas assay across six mTLS-positive lung tumors from patients with extreme ICI responses—three with an objective response and three with progressive disease (Figure 2A; Table S2). We strategically selected regions of interest based on TLS staining on consecutive slides by employing a combination of CD4/CD8/CD20/CD23 multiplex immunofluorescence (mIF) to identify mTLSs and CD45/PanCK morphologic markers to locate the tumor or stromal area. We further delineated mTLSs as whole areas of illumination (AOIs) and adjacent tumor tissue as stromal ($PanCK^-/CD45^{+/-}$) and tumor ($PanCK^+/CD45^-$) AOIs for comparison (Figure 2B; Table S3).

Comparative unsupervised clustering of significantly upregulated genes informed by Gene Ontology biological processes revealed distinct expression patterns in the stroma of responders versus non-responders.[17] In stark contrast, the mTLSs and tumor segments did not exhibit significant differential expression, suggesting a stroma's pivotal role in determining the response to immunotherapy in mTLS-positive NSCLC (Figure 2C). Specifically, genes encoding fibroblasts and components of the extracellular matrix were predominantly expressed in the stroma of non-responders, indicating a potential impact in resistance to immunotherapy (Figure 2D; Table S4).

Employing the SpatialDecon algorithm, we quantified the relative abundance of immune and stromal cell populations within each segment. Our analysis unveiled an enrichment of fibroblasts in the stroma of non-responders, providing further evidence for their involvement in immunotherapy resistance (Figure 2E). This enrichment was statistically significant, with a higher proportion of fibroblasts observed in the stroma of non-responders compared to responders ($p < 0.001$, Figure 2F). Furthermore, gene set enrichment analysis of Hallmark biological processes corroborated a pronounced expression of transforming growth factor β (TGF-β) signaling and epithelial-mesenchymal transition pathways in non-responders.[18] In

Figure 1. The presence of mTLSs is associated with clinical outcomes to immune checkpoint inhibitors in NSCLC

(A) Representative image field of immature and mature TLSs observed in two distinct tumor samples from an FFPE NSCLC adenocarcinoma section. Mature TLSs are defined by the presence of CD23-positive dendritic cells on IHC. The pictures correspond to H&E staining (left/middle column) and triple IHC staining of CD3/CD20/CD23 (with CD3, CD20, and CD23 stained in brown, purple, and green, respectively). The scale bars indicate 400 and 50 μm for the left and middle/right, respectively. Dashed lines delineate TLSs, and black cropped arrows highlight the tumor cells in the samples.

(B) Tissue-based genomic profiling landscape of NSCLC tumors according to TLS status ($N = 182$).

(C) Proportion of patients characterized by the absence or presence of either iTLSs or mTLSs according to the OS endpoint (OS < 24 months versus OS ≥ 24 months from treatment initiation). Statistical significance was determined by chi-squared test.

(D) Forest plot of multivariate Cox analysis of OS including baseline clinical and pathological features.

(E) Response rate, as defined per objective response (left) or RECIST 1.1 criteria (right), according to mTLS status: absence (negative, no TLSs or iTLSs) or presence (positive). Statistical significance was determined by chi-squared test.

(F) Kaplan-Meier analysis of the PFS of patients according to TLS status ($n = 509$; red curve: mTLS-enriched tumors; blue curve: mTLS-negative tumors). Numbers below each x axis indicate the number of patients at risk and those in parentheses are the number of events. Statistical significance was determined by log rank test.

(G) Kaplan-Meier analysis of the OS of patients according to TLS status ($n = 509$; red curve: mTLS-enriched tumors; blue curve: mTLS-negative tumors). Numbers below each x axis indicate the number of patients at risk and those in parentheses are the number of events. Statistical significance was determined by log rank test.

IHC, immunohistochemistry; H&E, hematoxylin and eosin; NSCLC, non-small cell lung carcinoma; NR, non-responder; OR, objective response; OS, overall survival; PFS, progression-free survival; PD, progressive disease; R, responder; SD, stable disease; TMB, tumor mutational burden.

See also Figures S1 and S2; Tables 1 and S1.

CellPress
OPEN ACCESS

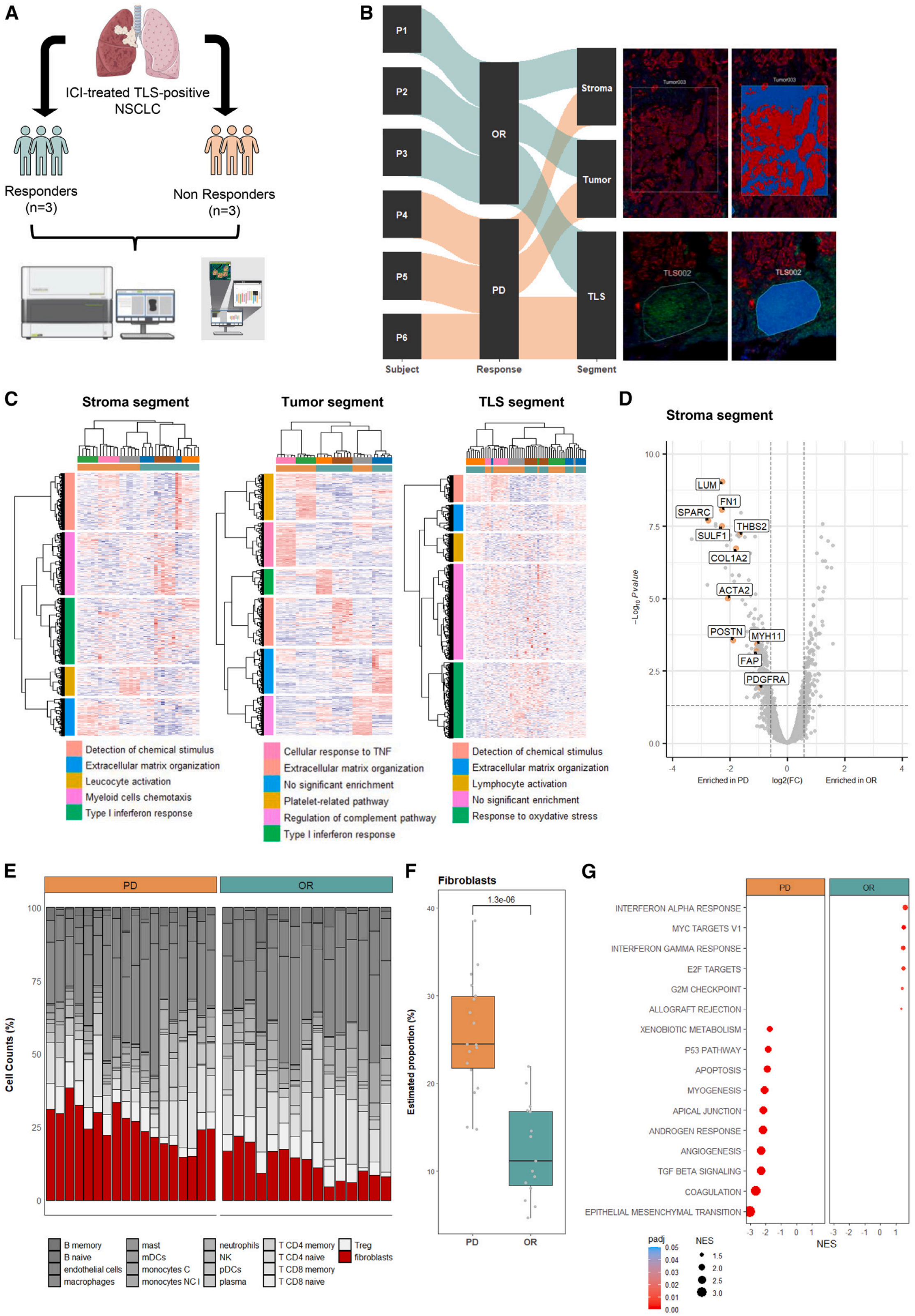

(legend on next page)

contrast, responders displayed significantly higher expression of pathways related to inflammatory processes, particularly those mediated by interferon α (IFN-α) and interferon γ (IFN-γ) (Figure 2G).

Stromal FAP$^+$αSMA$^+$ CAF and MYH11$^+$αSMA$^+$ CAF correlate with poor outcomes in ICI-treated mTLS-positive NSCLC

In pursuit of refining the predictive markers within the stroma of mTLS-positive NSCLC for ICI treatment response, we sought to validate the influence of distinct fibroblast populations at the histological level. Grounded in seminal findings from a recent study, which underscored the pivotal roles of fibroblast activation protein alpha (FAP)$^+$ alpha smooth muscle actin (αSMA)$^+$ cancer-associated fibroblast (CAF) and MYH11$^+$αSMA$^+$ CAF in NSCLC microenvironment, and considering the significant enrichment of *ACTA2*, *FAP*, and *MYH11* in non-responders to ICIs from previous spatial transcriptomic analysis (Table S4), we utilized a robust 5-color mIF panel.[19] This approach enabled the simultaneous detection of tumor cells, cytotoxic CD8$^+$ T cells, and these salient CAF subsets within the initial tumor samples of 77 patients (Figures 3A and S4A). Patient baseline characteristics are delineated in Table S5.

Our analysis revealed a higher stromal density of both FAP$^+$αSMA$^+$ and MYH11$^+$αSMA$^+$ CAFs in the non-responder group (Figures 3B–3E and S4A–S4E), suggesting that these CAF populations may be potential indicators of poor response to ICI therapy. The fraction of non-responders was significantly more pronounced among patients with high stromal FAP$^+$αSMA$^+$ CAF density (34/59, 57.6%) compared to those with low density (5/18, 27.8%; p = 0.027; Figure 3F), which also corresponded to a lower PFS (hazard ratio [HR]: 0.35; 95% CI: 0.17–0.72; p = 0.003; Figure 3G) and a non-significant trend toward reduced OS (p = 0.13; Figure 3H). A similar pattern was observed with MYH11$^+$αSMA$^+$ CAF density, with a higher proportion of non-responders (35/62, 56.5%) in the high-density category than in the low-density group (4/11, 36.4%; p = 0.038; Figure 3I), as well as shorter PFS (HR: 0.36; 95% CI: 0.16–0.79; p = 0.008; Figure 3J) and a trend toward decreased OS (p = 0.16; Figure 3K).

Moreover, the presence of both high FAP$^+$αSMA$^+$ CAF and MYH11$^+$αSMA$^+$ CAF correlated with even poorer outcomes, with these patients exhibiting significantly reduced response rates (p = 0.013; Figure S4F), PFS (HR: 0.35; 95% CI: 0.19–0.65; p < 0.001; Figure S4G), and OS (HR: 0.47; 95% CI: 0.23–0.94; p = 0.03; Figure S4H) compared to those with low CAF densities.

We further assessed the influence of these CAF subsets on the CD8$^+$ T cell distribution within the TME.[20] The density of stromal FAP$^+$αSMA$^+$ CAF was predominantly higher in tumors classified as "infiltrated" relative to those deemed "excluded" or "desert" (Figure S5A), with a significant fraction of infiltrated and excluded tumors being found in the FAP$^+$αSMA$^+$ CAF-high group (Figure S5B). Conversely, MYH11$^+$αSMA$^+$ CAF density was notably lower in infiltrated tumors when compared to excluded ones (Figure S5C), and excluded tumors were almost exclusively present in the MYH11$^+$αSMA$^+$ CAF-high group (Figure S5D).

FAP$^+$αSMA$^+$ CAF correlates with inflammatory response and exhaustion of CD8$^+$ T cells in the TME

We then focused on the interactions between the CD8$^+$ T cell infiltrate and the heterogeneous populations of CAFs. Through comprehensive bulk transcriptomic analysis of 40 mTLS-positive lung tumor samples, we identified distinct gene expression profiles (Figure 4A). The enrichment analysis of Hallmark pathways uncovered a significant association between FAP$^+$αSMA$^+$ CAF-high tumors and inflammatory pathways, including IFN-α, IFN-γ, and general inflammatory responses, suggesting a potential role of these CAFs in promoting an inflammatory TME (Figures 4B and 4C). Further gene set enrichment analysis emphasized that these same tumors were characterized by enhanced gene signatures of T cell exhaustion, which could undermine the cytotoxic activity of CD8$^+$ T cells and potentially diminish the efficacy of ICIs (Figures 4D and 4E).[21]

To substantiate our transcriptional insights with protein expression data, we performed an mIF analysis on 64 patients with mTLS-positive NSCLC using a 6-plex color panel for the markers CD8, PD1, CD39, LAG3, TIGIT, and TIM3 (Figure 4F). The patient characteristics for this subset are detailed in Table S8. The results consistently showed a greater density of intratumoral CD8$^+$PD1$^+$ T cells in the FAP$^+$αSMA$^+$ CAF-high tumors. These CD8$^+$ T cells also displayed heightened expression of exhaustion markers, suggesting a CAF-induced immunosuppressive state within the TME (Figures 4F and S6A). Interestingly, no discernible variation was found in the density of stromal CD8$^+$ T cells relative to the FAP$^+$αSMA$^+$ CAF status (Figure S6B), and no link between MYH11$^+$αSMA$^+$ CAF and T cell exhaustion was observed, indicating a unique influence of the FAP$^+$αSMA$^+$ CAF subset

Figure 2. The presence of fibroblasts in stroma of mTLS-positive NSCLC correlated with poor response to immune checkpoint inhibitors
(A) Tissue processing workflow for spatial transcriptomic of FFPE samples of TLS-positive NSCLC.
(B) Sankey plot and illustration of the distribution of selected AOIs. All scale bars, 50 μm.
(C) Unsupervised clustering heatmap of upregulated Gene Ontology (GO) pathways in stroma segment, tumor segment, and TLS segment, respectively.
(D) Volcano plot of differential gene expression between responders (PD, N = 3) and non-responders (OR, N = 3) in stroma segment.
(E) Stromal cell composition between non-responders (PD, N = 3) and responders (OR, N = 3) in stroma segment. A total of 18 AOIs in the PD group and 15 AOIs in the OR group are represented, respectively.
(F) Boxplot of estimated proportion of fibroblast population using SpatialDecon algorithm. p value was calculated using Wilcoxon test. Data are represented as median ± IQR.
(G) Bubble plot of Hallmark pathways analysis of the gene differentially expressed between responders (PD, N = 3) and non-responders (OR, N = 3) in stroma segment.
AOIs, areas of interest; FFPE, formalin-fixed, paraffin-embedded; ICIs, immune checkpoint inhibitors; IQR, interquartile range; NSCLC, non-small cell lung cancer; mTLSs, mature tertiary lymphoid structures.
See also Figure S3; Tables S2, S3, and S4.

CellPress
OPEN ACCESS

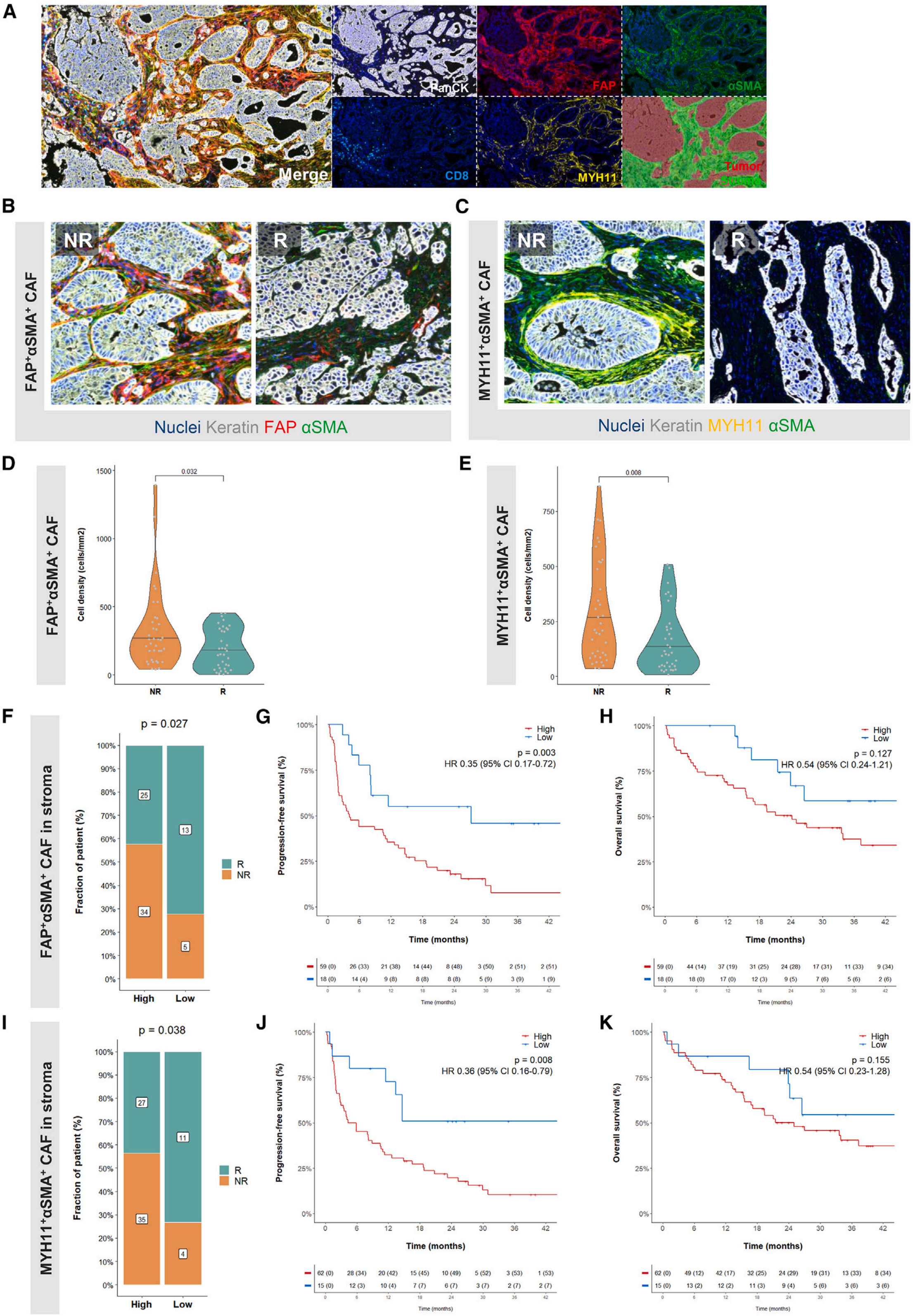

(legend on next page)

(Figure 4G). These findings lend support to the hypothesis that FAP^{+}αSMA^{+} CAFs contribute to a more inflammatory and may drive exhausted phenotype of CD8^{+} T cells in the TME.

MYH11^{+}αSMA^{+} CAF correlates with regulatory CD4^{+} T cell infiltration and immunosuppressive TME

Building upon prior evidence linking MYH11^{+}αSMA^{+} CAFs to altered T cell distribution within the TME,[19] our transcriptomic analysis further stratified the influence of these CAFs on the immune profile. Notably, the presence of MYH11^{+}αSMA^{+} CAFs appears to inversely correlate with immune-activating pathways such as those driven by tumor necrosis factor alpha and IFN-γ, which are significantly upregulated in tumors with low levels of these CAFs, as depicted in Figures 5A and 5B. This suggests that MYH11^{+}αSMA^{+} CAF-low tumors may harbor a more conducive microenvironment for antitumor immunity.

Gene set enrichment analyses revealed a stark contrast in tumors with high MYH11^{+}αSMA^{+} CAF density, unveiling a significant enrichment of regulatory T cell (Treg) gene signatures, known contributors to immunosuppression within the TME and impediments to the success of immune checkpoint inhibition therapies (Figures 5C and 5D).[22] The mIF-based profiling of a cohort of 64 NSCLC samples with a 6-color CD4, CD8, CD20, FoxP3, inducible T cell costimulator (ICOS), and T cell immunoreceptor with immunoglobulin (Ig) and ITIM domains (TIGIT) panel further substantiated these findings, revealing a heightened stromal CD4^{+} T cell presence in MYH11^{+}αSMA^{+} CAF-high tumors, without comparable changes in other T or B cell subsets (Figures S6C and S6D).

A more detailed analysis of the MYH11^{+}αSMA^{+} CAF-high group uncovered an increased infiltration of stromal regulatory CD4^{+}FoxP3^{+} T cells, which notably expressed high levels of the immunosuppressive markers TIGIT and ICOS (Figure 5E), signifying a robustly immunosuppressive phenotype. In contrast, no such association was detected between stromal-infiltrating Tregs and the FAP^{+}αSMA^{+} CAF category (Figure 5F), suggesting the singular role of MYH11^{+}αSMA^{+} CAFs in shaping the immune milieu.

Further emphasizing the immunosuppressive nature of MYH11^{+}αSMA^{+} CAF-high tumors, we observed an elevated ratio of stromal CD4^{+}FoxP3^{+} T cells to CD8^{+} T cells in these samples, compared to those with mid or low levels of MYH11^{+}αSMA^{+} CAFs (Figure 5G). This ratio did not show a correlation with the FAP^{+}αSMA^{+} CAF profiles (Figure 5H). Corroboratively, the classification of FAP^{+}αSMA^{+} CAF and MYH11^{+}αSMA^{+} CAF through mIF aligned with the transcriptomic signatures associated with the respective CAF subtype, its ligands, and extracellular matrix composition, further detailed in Figures S6E and S6F. Collectively, these results underscore the impact of MYH11^{+}αSMA^{+} CAFs on fostering an immunosuppressive TME, marked by increased Treg infiltration, in mTLS-positive NSCLC.

DISCUSSION

We have previously reported that mTLS presence within the TME correlates with improved outcomes in patients undergoing immunotherapy, across several tumor types including those typically resistant to ICIs, such as sarcomas.[14] Our present study underscores the importance of mTLSs as predictive markers for the response to PD1/PD-L1 blockade in NSCLC, highlighting their role in driving an effective antitumor immune response. However, our investigation, along with others examining the predictive role of TLSs in NSCLC, identified that a significant subset of patients with mTLS-positive NSCLC does not derive benefit from immune checkpoint blockade.[15,16,23] Unraveling the mechanisms behind immunotherapy resistance in mTLS-positive NSCLC is crucial for pinpointing factors that limit the success of immunotherapeutic strategies in these patients.

In many tumors types, including NSCLC, CAFs represent the main constituents of the tumor-surrounding stroma that impact treatment efficacy.[24,25] Through comparative spatial transcriptomics of ICI-treated patients exhibiting extreme responses (long-lasting response versus primary resistance), our study revealed that the key difference between these two phenotypes lies in the prevalence of specific CAF populations within the NSCLC microenvironment. Further validation of these

Figure 3. Stromal FAP^{+}αSMA^{+} CAF and MYH11^{+}αSMA^{+} CAF correlate with clinical outcome in patients treated with immune checkpoint inhibitors

(A) Representative image field of PanCK/CD8/FAP/MYH11/αSMA/DAPI multiplexed immunohistofluorescence panel on an FFPE NSCLC adenocarcinoma section. Illustration of the segmentation strategy of the tissue in "stroma" and "tumor" areas is shown at the bottom right. All scale bars, 200 μm.

(B) Representative image field of FAP^{+}αSMA^{+} CAF infiltration in the tumor microenvironment of non-responders (NR, left) and responders (R, right) to ICI. All scale bars, 50 μm.

(C) Representative image field of MYH11^{+}αSMA^{+} CAF infiltration in the tumor microenvironment of non-responders (NR, left) and responders (R, right) to ICI. All scale bars, 50 μm.

(D) Density of FAP^{+}αSMA^{+} CAF in the stroma areas of non-responders and responders to ICI. The p values were calculated using Wilcoxon tests. Data are represented as median.

(E) Density of MYH11^{+}αSMA^{+} CAF in the stroma areas of non-responders and responders to ICI. The p values were calculated using Wilcoxon tests. Data are represented as median.

(F) Proportion of patients with high and low density of FAP^{+}αSMA^{+} CAF according to response. The p value was calculated using an χ^2 test.

(G) Kaplan-Meier curves of the PFS of patients classified as high or low based on levels of stromal FAP^{+}αSMA^{+} CAF.

(H) Kaplan-Meier curves of the OS of patients classified as high or low based on levels of stromal FAP^{+}αSMA^{+} CAF.

(I) Proportion of patients with high and low density of FAP^{+}αSMA^{+} CAF according to response. The p value was calculated using an χ^2 test.

(J) Kaplan-Meier curves of the PFS of patients classified as high or low based on levels of stromal MYH11^{+}αSMA^{+} CAF.

(K) Kaplan-Meier curves of the OS of patients classified as high or low based on levels of stromal MYH11^{+}αSMA^{+} CAF.

CAF, cancer-associated fibroblast; ICI, immune checkpoint inhibitors; NR, non-responder; OS, overall survival; PFS, progression-free survival; R, responder; TLSs, tertiary lymphoid structures.

See also Figures S4 and S5; Table S5.

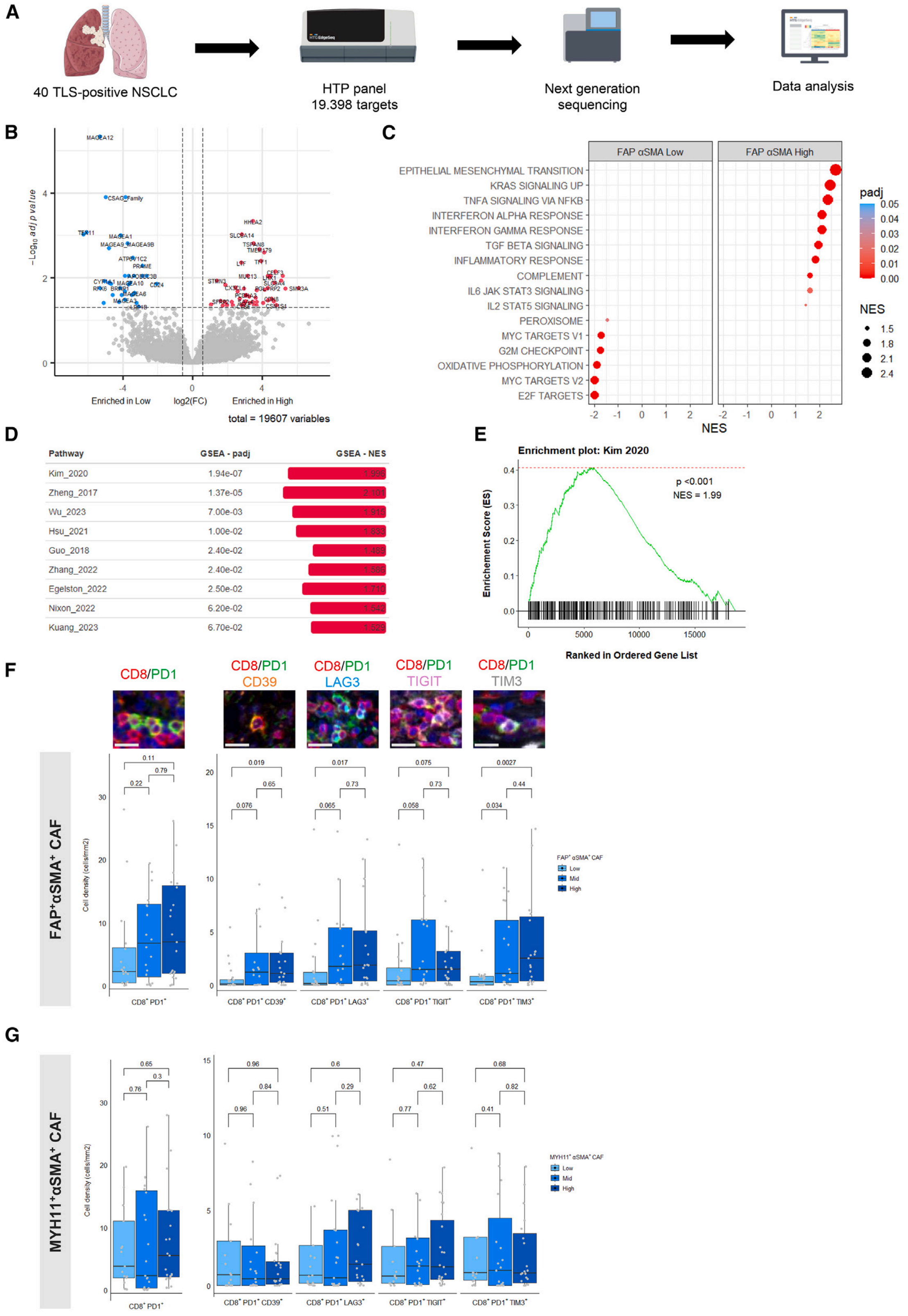

(legend on next page)

transcriptomic findings through mIHF confirmed that, specifically, FAP$^+$αSMA$^+$ CAFs and MYH11$^+$αSMA$^+$ CAFs are linked to poor outcomes in patients with mTLS-positive NSCLC treated with ICIs, suggesting their role in promoting an environment that supports tumor progression and therapy resistance. In line with these findings, transcriptional signature analysis of breast cancer identified distinct CAF clusters that associated with primary immunotherapy resistance.[26]

Previous studies have demonstrated that fibroblasts play a crucial role in the architecture and functionality of cancer-associated TLSs.[27,28] Indeed, fibroblasts within cancer-associated TLSs and non-structured lymphoid aggregates perform functions similar to those of lymphoid tissue organizer (LTo) cells and fibroblastic reticular cells (FRCs) observed in non-cancerous, chronically inflamed tissues. This transformation of local fibroblasts into LTo-like and subsequently into cytokine and chemokine-producing FRC-like cells is vital for TLS formation. Notably, preclinical studies have shown that fibroblasts orchestrating TLS formation in malignant tumors such as melanoma or colorectal cancer are exclusively FAP negative.[28] Recently, the actin regulatory protein isoform hMENA/hMENAΔv6 was found to be expressed by pro-tumoral FAP-positive CAFs in NSCLC cells. This isoform contributes to inhibiting the LTβR-related nuclear factor κB signaling pathway, reducing CXCL13 secretion, and promoting fibronectin production, ultimately impairing the formation of TLSs and the response to ICIs.[29] Collectively, these findings provided insight into the mechanisms used by the CAF, highlighting a coordinated interaction between CAFs and tumor-associated TLSs.

Phenotypic and spatial features of CAF are heterogeneous in NSCLC.[24,30] Our findings indicate that the presence of two distinct fibroblast populations, namely FAP$^+$αSMA$^+$ CAFs and MYH11$^+$αSMA$^+$ CAFs, is associated with adverse outcomes in patients with mTLS-positive NSCLC undergoing ICI treatment. These fibroblast populations have been recently described in studies using single-cell RNA sequencing alongside various imaging techniques to analyze the NSCLC microenvironment.[19] FAP$^+$SMA$^+$ CAFs have been described to form extensive layers within the stromal regions or around tumor cell clusters in late-stage tumors, while MYH11$^+$SMA$^+$ CAFs were predominantly found in early-stage tumors, encircling clusters of cancer cells in a single layer. Consistent with our observations, a spatially resolved single-cell imaging mass cytometry analysis of lung tumors identified 11 CAF phenotypes and revealed that the SMA$^+$ CAF subset, which encompasses both FAP$^+$αSMA$^+$ CAF and MYH11$^+$SMA$^+$ CAF populations, made up the biggest proportion of CAFs and was found almost exclusively within the stromal compartment.[25]

Our gene expression profiling and multiplex spatial imaging of tumor specimens, along with the analysis of spatial relationships among CAFs and immune cells, revealed a significant increase in the proportion of immune-excluded tumors among mTLS-positive NSCLC with high-density levels of FAP$^+$αSMA$^+$ CAFs and MYH11$^+$SMA$^+$ CAFs. This suggests that these fibroblast populations significantly impact CD8$^+$ T cell trafficking as previously shown by Grout et al. in a recent report of fibroblast landscape in NSCLC.[19] In agreement with this latter evidence, the SMA$^+$ CAF group showed spatial localization near vessel structures and supported an immune-enriched microenvironment, suggesting that FAP$^+$αSMA$^+$ CAFs and MYH11$^+$SMA$^+$ CAFs actively promote immune cell infiltration.[25]

Furthermore, we discovered that tumors rich in FAP$^+$SMA$^+$ CAFs also exhibit higher levels of CD8$^+$ T cell exhaustion. This finding aligns with recent research investigating how CAFs influence the phenotype and function of tumor-infiltrating lymphocytes (TILs) in NSCLC. This study found not only an increased expression of PD1 and T cell Ig and mucin-domain containing-3 (TIM3) but also a significant rise in CD39 levels on T cells when co-cultured with CAFs, a process driven by TGF-β.[31] Preclinical experiments in human NSCLC models have shown that CAFs deliver complex regulatory signals to TIL CD8$^+$ T cells, thereby suppressing their antitumor activity.[32] Interestingly, FAP$^+$ CAF-mediated desmoplastic stroma demonstrated to restrict T cell extravasation, mediate immune exclusion, and alter CD8$^+$ T cell function.[33] These findings are in line with evidences that antitumor immune response to PD1/PD-L1 blockade is attenuated by TGF-β-associated CAF and immune evasion.[34,35] These results highlight the impact of CAFs in promoting T cell-mediated immunosuppressive TME.

In our study, the presence of MYH11$^+$αSMA$^+$ CAFs was particularly associated with an increased abundance of CD4$^+$ Treg cells in the TME. In humans, Treg cells, known for their high expression of FoxP3, play a crucial role in suppressing antitumor immunity.[36,37] In addition, recent evidence suggested that PD1 blockade enhances the suppressive activity of Treg cells that express high levels of PD1.[38] Our team has previously demonstrated that Treg cells are more abundant in TLS-positive sarcomas in patients resistant to immunotherapy compared to

Figure 4. FAP$^+$αSMA$^+$ CAF correlates with inflammatory response and exhaustion of CD8 T cells in the tumor microenvironment
(A) Tissue processing workflow for regional transcriptomic of FFPE samples of TLS-positive NSCLC (n = 40).
(B) Volcano plot of the differentially expressed gene between FAP$^+$αSMA$^+$ CAF-high (N = 28) and FAP$^+$αSMA$^+$ CAF-low (N = 12) patients.
(C) Bubble plot of Hallmark pathway analysis of the differentially expressed genes between FAP$^+$αSMA$^+$ CAF-high (N = 28) and FAP$^+$αSMA$^+$ CAF-low (N = 12) patients.
(D) GSEA analysis of exhaustion T cell gene signatures.
(E) GSEA plot of exhaustion T cell pathway using Kim et al. signature.
(F) Representative image field and corresponding density of intratumoral CD8$^+$PD1$^+$, CD8$^+$PD1$^+$CD39$^+$, CD8$^+$PD1$^+$LAG3$^+$, CD8$^+$PD1$^+$TIGIT$^+$, and CD8$^+$PD1$^+$TIM3$^+$ T cells according to stromal FAP$^+$αSMA$^+$ CAF category. The p values were calculated using Wilcoxon tests. All scale bars, 20 μm. Data are represented as median ± IQR.
(G) Density of intratumoral CD8$^+$PD1$^+$, CD8$^+$PD1$^+$CD39$^+$, CD8$^+$PD1$^+$LAG3$^+$, CD8$^+$PD1$^+$TIGIT$^+$, and CD8$^+$PD1$^+$TIM3$^+$ T cells according to stromal MYH11$^+$αSMA$^+$ CAF category. The p values were calculated using Wilcoxon tests. Data are represented as median ± IQR. CAF, cancer-associated fibroblast; GSEA, gene set enrichment analysis; IQR, interquartile range; NES, normalized enrichment score.
See also Figure S6; Tables S6 and S8.

CellPress
OPEN ACCESS

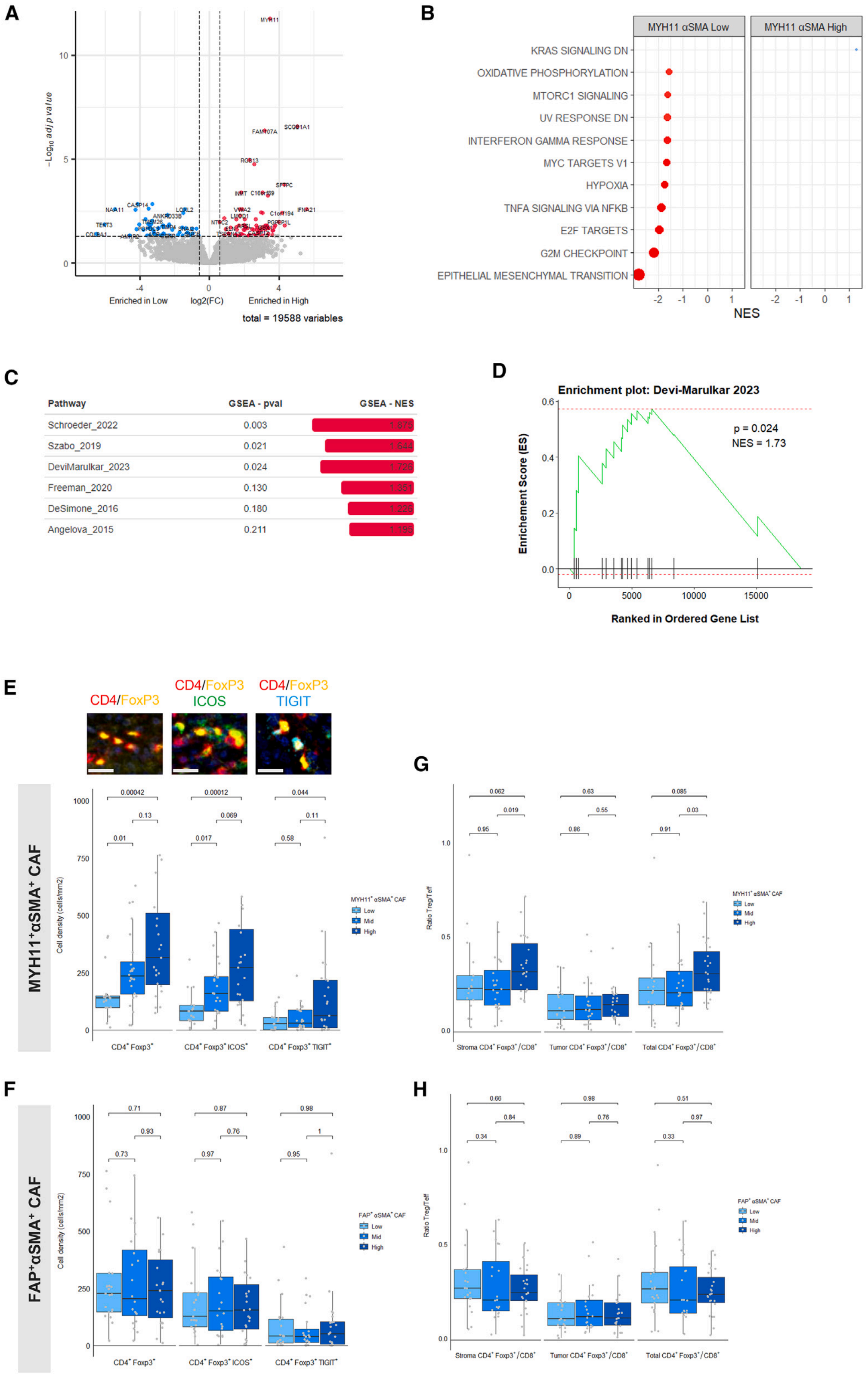

Pathway	GSEA - pval	GSEA - NES
Schroeder_2022	0.003	1.875
Szabo_2019	0.021	1.644
DeviMarulkar_2023	0.024	1.726
Freeman_2020	0.130	1.351
DeSimone_2016	0.180	1.226
Angelova_2015	0.211	1.195

(legend on next page)

those who are responsive.[39] Previous research has shown that CAFs can promote the migration and enhance the presence of Treg cells across various tumor types, such as colorectal and breast cancer.[40–43] Our findings indicate that this specific population of MYH11⁺αSMA⁺ CAFs may also exist in patients with advanced NSCLC, potentially adversely affecting the TME by recruiting Treg cells.

Despite the valuable insights gained from this study, it is important to acknowledge several limitations. First, the absence of suitable preclinical models, including those for FAP⁺αSMA⁺ and MYH11⁺αSMA⁺ CAFs and immunotherapy-sensitive lung cancer with mTLSs, hindered our ability to conduct functional experiments to definitively establish a causal relationship between these specific CAF subsets, T cell exhaustion markers, and Treg infiltration. Second, to gain a more comprehensive understanding, future studies should explore the impact of these CAF subsets on B cell subsets and humoral responses in the context of mTLS-positive tumors. Third, our study did not include enough patients who received chemotherapy or immunotherapy in TLS-positive or TLS-negative lung tumors to draw definitive conclusions about the prognostic and predictive value of these specific CAF subsets in these scenarios. A better understanding of the biology and function of CAFs is essential for advancing the development of novel immunotherapy strategies tailored to patients with lung cancer.

In summary, our findings recognize mTLSs as a dependable biomarker for identifying patients with NSCLC who are more likely to respond positively to ICIs. This specific patient group may be prime candidates for a chemotherapy-free regimen to circumvent the adverse effects associated with platinum-based chemotherapy. When delving into the reasons behind the resistance to immunotherapy in mTLS-positive NSCLC, it becomes apparent that mitigating the detrimental influence of certain CAF populations could significantly enhance the effectiveness of immunotherapy for patients with mTLS-positive NSCLC. Although attempts to directly target fibroblasts, such as using FAP-directed agents, have not met with success thus far, exploring alternative tactics that counteract the pro-tumoral activities of fibroblasts in mTLS-positive NSCLC is warranted.[44] A promising strategy could be to more accurately target the exhaustion of CD8⁺ T cells through the inhibition of various immune checkpoints. This method will be explored in the forthcoming INDIGO study, which aims to assess the combination of the PD-L1 inhibitor, atezolizumab and of the anti-TIGIT, tiragolumab as a first-line treatment for patients with advanced mTLS-positive PD-L1-low NSCLC.

Limitations of the study

While this study provides valuable insights, it is important to acknowledge several limitations. First of all, the sample size in this study was insufficient to allow for a comprehensive analysis of the prognostic and predictive significance of CAF subsets in relation to different treatment modalities (i.e., chemotherapy or immunotherapy) and TLS status. In addition, the limitations imposed by the availability of accurate preclinical models precluded the performance of functional experiments to establish a causal relationship. Finally, a deeper understanding of the interactions between CAFs and B cell subsets is crucial for elucidating the role of these cells in the TME of TLS-positive NSCLC and the development of effective immunotherapies.

RESOURCE AVAILABILITY

Lead contact

Further information and requests for resources and reagents should be directed to and will be fulfilled by the lead contact, Antoine Italiano (a.italiano@bordeaux.unicancer.fr).

Materials availability

This study did not generate new unique reagents.

Data and code availability

(1) The datasets and code that support the findings of this study are not publicly available due to information that could compromise research participant consent.
(2) According to French/European regulations, any reuse of the data must be approved by the ethics committee “CPP du Sud-Ouest et d’ Outre-Mer III,” Bordeaux, France. Each request for access to the clinical dataset, spatial and regional transcriptomic datasets, and immunohistochemistry and immunofluorescence datasets (including the images) that underlie the results reported in this article will be granted after a request is sent to the corresponding author (A.I.) and approval by the ethics committee.
(3) Individual participant data will be available after deidentification to researchers who provide a methodologically sound proposal. Proposals may be submitted up to 36 months following article publication.
(4) Any additional information required to reanalyze the data reported in this work paper is available from the lead contact (A.I.) upon request.

Figure 5. MYH11⁺αSMA⁺ CAF correlates with regulatory CD4 T cell infiltration and immunosuppressive tumor microenvironment
(A) Volcano plot of the differentially expressed gene between MYH11⁺αSMA⁺ CAF-high (N = 31) and MYH11⁺αSMA⁺ CAF-low (N = 9) patients.
(B) Hallmark pathway analysis of the differentially expressed genes between MYH11⁺αSMA⁺ CAF-high (N = 31) and MYH11⁺αSMA⁺ CAF-low (N = 9) patients.
(C) GSEA analysis of regulatory T cell gene signatures.
(D) GSEA plot of regulatory T cell pathway using Devi-Marulkar et al. signature.
(E) Representative image field and corresponding density of stromal CD4⁺Foxp3⁺, CD4⁺Foxp3⁺ICOS⁺, and CD4⁺Foxp3⁺TIGIT⁺ T cells according to stromal MYH11⁺αSMA⁺ CAF category. The p values were calculated using Wilcoxon tests. All scale bars, 20 μm. Data are represented as median ± IQR.
(F) Density of stromal CD4⁺Foxp3⁺, CD4⁺Foxp3⁺ICOS⁺, and CD4⁺Foxp3⁺TIGIT⁺ T cells according to stromal FAP⁺αSMA⁺ CAF category. The p values were calculated using Wilcoxon tests. Data are represented as median ± IQR.
(G) Stromal CD4⁺Foxp3⁺/CD8⁺ T cell ratio, intratumoral CD4⁺Foxp3⁺/CD8⁺ T cell ratio, and total CD4⁺Foxp3⁺/CD8⁺ T cell ratio according to stromal MYH11⁺αSMA⁺ CAF category. The p values were calculated using Wilcoxon tests. Data are represented as median ± IQR.
(H) Stromal CD4⁺Foxp3⁺/CD8⁺ T cell ratio, intratumoral CD4⁺Foxp3⁺/CD8⁺ T cell ratio, and total CD4⁺Foxp3⁺/CD8⁺ T cell ratio according to stromal FAP⁺αSMA⁺ CAF category. The p values were calculated using Wilcoxon tests. Data are represented as median ± IQR. CAF, cancer-associated fibroblast; IQR, interquartile range.
See also Figure S6; Tables S7 and S8.

ACKNOWLEDGMENTS

We thank the important contributions of patients who participated in this study.

The computational work was supported by Explicyte Immuno-oncology, Bordeaux, France.

We thank the department of medical oncology and the pathology core of Institut Bergonié (Bordeaux, France), the department of oncology of the Centre Hospitalier de la Côte Basque (Bayonne, France), Atlantic Pathology (Saint Pierre d'Irrube, France), and the department of oncology of the Clinique Marzet (Pau, France) for their support and their contribution to obtaining and studying additional FFPE NSCLC samples.

This research was a collaborative effort of the imCORE Network, made possible through support from F. Hoffmann-La Roche.

AUTHOR CONTRIBUTIONS

F.P.: conceptualization, data curation, formal analysis, investigation, and writing – original draft; J.-P.G.: conceptualization, data curation, formal analysis, methodology, writing – original draft, and editing; C.R.: formal analysis; O.L.: formal analysis; O.O.: formal analysis; M.D.C.: resources; L.V.: investigation; J.-M.C.: investigation; E.C.: investigation; M.B.: investigation; T.G.: resources; A.T.: investigation; S.L.M.: resources; R.J.J.: writing – review; A.B.: conceptualization, supervision, methodology, and writing – review and editing; A.I.: conceptualization, supervision, funding acquisition, methodology, and writing – review and editing. All authors were involved in critical review of the manuscript and approved the submitted version.

DECLARATION OF INTERESTS

F.P., J.-P.G., C.R., O.L., O.O., and A.B. are employees of Explicyte. R.J.J. is an employee and stockholder of Roche/Genentech. A.I. received research grants from AstraZeneca, Bayer, BMS, Chugai, Merck, MSD, Pharmamar, Novartis, and Roche and personal fees from Epizyme, Bayer, Deciphera, Lilly, Parthenon, Roche, and Springworks.

STAR★METHODS

Detailed methods are provided in the online version of this paper and include the following:

- KEY RESOURCES TABLE
- EXPERIMENTAL MODEL AND STUDY PARTICIPANT DETAILS
  - Patient population
  - Treatments and evaluation
- METHOD DETAILS
  - Immunohistochemistry (IHC) stainings and TLS assessment
  - Genomic, tumor mutation burden (TMB) and microsatellite instability (MSI) analysis
  - Spatial transcriptomics
  - Multiplex immunohistofluorescence assay
  - Quantitative analysis of T cells infiltrate (immune contexture)
  - Regional HTG transcriptomic analysis
- QUANTIFICATION AND STATISTICAL ANALYSIS
- ADDITIONAL RESOURCES

SUPPLEMENTAL INFORMATION

Supplemental information can be found online at https://doi.org/10.1016/j.xcrm.2025.101934.

Received: July 9, 2024
Revised: September 13, 2024
Accepted: January 8, 2025
Published: February 4, 2025

REFERENCES

1. Siegel, R.L., Giaquinto, A.N., and Jemal, A. (2024). Cancer statistics, 2024. CA. Cancer J. Clin. *74*, 12–49.
2. Wang, M., Herbst, R.S., and Boshoff, C. (2021). Toward personalized treatment approaches for non-small-cell lung cancer. Nat. Med. *27*, 1345–1356.
3. Reck, M., Rodríguez-Abreu, D., Robinson, A.G., Hui, R., Csőszi, T., Fülöp, A., Gottfried, M., Peled, N., Tafreshi, A., Cuffe, S., et al. (2016). Pembrolizumab versus Chemotherapy for PD-L1–Positive Non–Small-Cell Lung Cancer. N. Engl. J. Med. *375*, 1823–1833.
4. Gettinger, S., Horn, L., Jackman, D., Spigel, D., Antonia, S., Hellmann, M., Powderly, J., Heist, R., Sequist, L.V., Smith, D.C., et al. (2018). Five-Year Follow-Up of Nivolumab in Previously Treated Advanced Non-Small-Cell Lung Cancer: Results From the CA209-003 Study. J. Clin. Oncol. *36*, 1675–1684.
5. Gandhi, L., Rodríguez-Abreu, D., Gadgeel, S., Esteban, E., Felip, E., De Angelis, F., Domine, M., Clingan, P., Hochmair, M.J., Powell, S.F., et al. (2018). Pembrolizumab plus Chemotherapy in Metastatic Non-Small-Cell Lung Cancer. N. Engl. J. Med. *378*, 2078–2092.
6. Garon, E.B., Hellmann, M.D., Rizvi, N.A., Carcereny, E., Leighl, N.B., Ahn, M.-J., Eder, J.P., Balmanoukian, A.S., Aggarwal, C., Horn, L., et al. (2019). Five-Year Overall Survival for Patients With Advanced Non−Small-Cell Lung Cancer Treated With Pembrolizumab: Results From the Phase I KEYNOTE-001 Study. J. Clin. Oncol. *37*, 2518–2527.
7. Herbst, R.S., Giaccone, G., de Marinis, F., Reinmuth, N., Vergnenegre, A., Barrios, C.H., Morise, M., Felip, E., Andric, Z., Geater, S., et al. (2020). Atezolizumab for First-Line Treatment of PD-L1–Selected Patients with NSCLC. N. Engl. J. Med. *383*, 1328–1339.
8. Hendriks, L.E., Kerr, K.M., Menis, J., Mok, T.S., Nestle, U., Passaro, A., Peters, S., Planchard, D., Smit, E.F., Solomon, B.J., et al. (2023). Non-oncogene-addicted metastatic non-small-cell lung cancer: ESMO Clinical Practice Guideline for diagnosis, treatment and follow-up. Ann. Oncol. *34*, 358–376.
9. Camidge, D.R., Doebele, R.C., and Kerr, K.M. (2019). Comparing and contrasting predictive biomarkers for immunotherapy and targeted therapy of NSCLC. Nat. Rev. Clin. Oncol. *16*, 341–355.
10. Horvath, L., Thienpont, B., Zhao, L., Wolf, D., and Pircher, A. (2020). Overcoming immunotherapy resistance in non-small cell lung cancer (NSCLC) - novel approaches and future outlook. Mol. Cancer *19*, 141.
11. Ricciuti, B., Lamberti, G., Puchala, S.R., Mahadevan, N.R., Lin, J.-R., Alessi, J.V., Chowdhury, A., Li, Y.Y., Wang, X., Spurr, L., et al. (2024). Genomic and Immunophenotypic Landscape of Acquired Resistance to PD-(L)1 Blockade in Non-Small-Cell Lung Cancer. J. Clin. Oncol. *42*, 1311–1321.
12. Fridman, W.H., Meylan, M., Petitprez, F., Sun, C.-M., Italiano, A., and Sautès-Fridman, C. (2022). B cells and tertiary lymphoid structures as determinants of tumour immune contexture and clinical outcome. Nat. Rev. Clin. Oncol. *19*, 441–457.
13. Schumacher, T.N., and Thommen, D.S. (2022). Tertiary lymphoid structures in cancer. Science *375*, eabf9419.
14. Vanhersecke, L., Brunet, M., Guégan, J.-P., Rey, C., Bougouin, A., Cousin, S., Moulec, S.L., Besse, B., Loriot, Y., Larroquette, M., et al. (2021). Mature tertiary lymphoid structures predict immune checkpoint inhibitor efficacy in solid tumors independently of PD-L1 expression. Nat. Cancer *2*, 794–802.
15. Brunet, M., Crombé, A., Cousin, S., Vanhersecke, L., Le Loarer, F., Bessede, A., and Italiano, A. (2022). Mature tertiary lymphoid structure is a specific biomarker of cancer immunotherapy and does not predict outcome to chemotherapy in non-small-cell lung cancer. Ann. Oncol. Off. J. Eur. Soc. Med. Oncol. *33*, 1084–1085.
16. Patil, N.S., Nabet, B.Y., Müller, S., Koeppen, H., Zou, W., Giltnane, J., Au-Yeung, A., Srivats, S., Cheng, J.H., Takahashi, C., et al. (2022).

Intratumoral plasma cells predict outcomes to PD-L1 blockade in non-small cell lung cancer. Cancer Cell *40*, 289–300.e4.

17. Gene Ontology Consortium, Aleksander, S.A., Balhoff, J., Carbon, S., Cherry, J.M., Drabkin, H.J., Ebert, D., Feuermann, M., Gaudet, P., Harris, N.L., et al. (2023). The Gene Ontology knowledgebase in 2023. Genetics *224*, iyad031.
18. Liberzon, A., Birger, C., Thorvaldsdóttir, H., Ghandi, M., Mesirov, J.P., and Tamayo, P. (2015). The Molecular Signatures Database (MSigDB) hallmark gene set collection. Cell Syst. *1*, 417–425.
19. Grout, J.A., Sirven, P., Leader, A.M., Maskey, S., Hector, E., Puisieux, I., Steffan, F., Cheng, E., Tung, N., Maurin, M., et al. (2022). Spatial Positioning and Matrix Programs of Cancer-Associated Fibroblasts Promote T-cell Exclusion in Human Lung Tumors. Cancer Discov. *12*, 2606–2625.
20. Bruni, D., Angell, H.K., and Galon, J. (2020). The immune contexture and Immunoscore in cancer prognosis and therapeutic efficacy. Nat. Rev. Cancer *20*, 662–680.
21. Chow, A., Perica, K., Klebanoff, C.A., and Wolchok, J.D. (2022). Clinical implications of T cell exhaustion for cancer immunotherapy. Nat. Rev. Clin. Oncol. *19*, 775–790.
22. Tay, C., Tanaka, A., and Sakaguchi, S. (2023). Tumor-infiltrating regulatory T cells as targets of cancer immunotherapy. Cancer Cell *41*, 450–465.
23. Srivastava, M.K., Gayevskiy, V., Ma, V., Estay, I., Rodas, M.L.d., Rajendran, B.K., Hoang, T., Ballinger, M., Amin, R., Herbst, R.S., et al. (2023). 606 IMpower110: Tertiary lymphoid structures (TLS) and clinical outcomes in advanced non-small cell lung cancer (NSCLC) treated with first-line atezolizumab or chemotherapy. J Immunother Cancer *11*, A690. https://doi.org/10.1136/jitc-2023-SITC2023.0606.
24. Caligiuri, G., and Tuveson, D.A. (2023). Activated fibroblasts in cancer: Perspectives and challenges. Cancer Cell *41*, 434–449.
25. Cords, L., Engler, S., Haberecker, M., Rüschoff, J.H., Moch, H., de Souza, N., and Bodenmiller, B. (2024). Cancer-associated fibroblast phenotypes are associated with patient outcome in non-small cell lung cancer. Cancer Cell *42*, 396–412.e5.
26. Kieffer, Y., Hocine, H.R., Gentric, G., Pelon, F., Bernard, C., Bourachot, B., Lameiras, S., Albergante, L., Bonneau, C., Guyard, A., et al. (2020). Single-Cell Analysis Reveals Fibroblast Clusters Linked to Immunotherapy Resistance in Cancer. Cancer Discov. *10*, 1330–1351.
27. Barone, F., Gardner, D.H., Nayar, S., Steinthal, N., Buckley, C.D., and Luther, S.A. (2016). Stromal Fibroblasts in Tertiary Lymphoid Structures: A Novel Target in Chronic Inflammation. Front. Immunol. *7*, 477.
28. Rodriguez, A.B., Peske, J.D., Woods, A.N., Leick, K.M., Mauldin, I.S., Meneveau, M.O., Young, S.J., Lindsay, R.S., Melssen, M.M., Cyranowski, S., et al. (2021). Immune mechanisms orchestrate tertiary lymphoid structures in tumors via cancer-associated fibroblasts. Cell Rep. *36*, 109422.
29. Modugno, F.D., Carlo, A.D., Spada, S., Palermo, B., D'Ambrosio, L., D'Andrea, D., Morello, G., Belmonte, B., Sperduti, I., Balzano, V., et al. (2024). Tumoral and stromal hMENA isoforms impact tertiary lymphoid structure localization in lung cancer and predict immune checkpoint blockade response in patients with cancer. EBioMedicine *101*, 105003. https://doi.org/10.1016/j.ebiom.2024.105003.
30. Mathieson, L., Koppensteiner, L., Dorward, D.A., O'Connor, R.A., and Akram, A.R. (2024). Cancer-associated fibroblasts expressing fibroblast activation protein and podoplanin in non-small cell lung cancer predict poor clinical outcome. Br. J. Cancer *130*, 1758–1769.
31. O'Connor, R.A., Chauhan, V., Mathieson, L., Titmarsh, H., Koppensteiner, L., Young, I., Tagliavini, G., Dorward, D.A., Prost, S., Dhaliwal, K., et al. (2021). T cells drive negative feedback mechanisms in cancer associated fibroblasts, promoting expression of co-inhibitory ligands, CD73 and IL-27 in non-small cell lung cancer. OncoImmunology *10*, 1940675.
32. Nazareth, M.R., Broderick, L., Simpson-Abelson, M.R., Kelleher, R.J., Jr., Yokota, S.J., and Bankert, R.B. (2007). Characterization of Human Lung Tumor-Associated Fibroblasts and Their Ability to Modulate the Activation of Tumor-Associated T Cells1. J. Immunol. *178*, 5552–5562.
33. Xiao, Z., Todd, L., Huang, L., Noguera-Ortega, E., Lu, Z., Huang, L., Kopp, M., Li, Y., Pattada, N., Zhong, W., et al. (2023). Desmoplastic stroma restricts T cell extravasation and mediates immune exclusion and immunosuppression in solid tumors. Nat. Commun. *14*, 5110.
34. Chakravarthy, A., Khan, L., Bensler, N.P., Bose, P., and De Carvalho, D.D. (2018). TGF-β-associated extracellular matrix genes link cancer-associated fibroblasts to immune evasion and immunotherapy failure. Nat. Commun. *9*, 4692.
35. Mariathasan, S., Turley, S.J., Nickles, D., Castiglioni, A., Yuen, K., Wang, Y., Kadel, E.E., III, Koeppen, H., Astarita, J.L., Cubas, R., et al. (2018). TGFβ attenuates tumour response to PD-L1 blockade by contributing to exclusion of T cells. Nature *554*, 544–548.
36. Wing, J.B., Tanaka, A., and Sakaguchi, S. (2019). Human FOXP3+ Regulatory T Cell Heterogeneity and Function in Autoimmunity and Cancer. Immunity *50*, 302–316.
37. Kumagai, S., Itahashi, K., and Nishikawa, H. (2024). Regulatory T cell-mediated immunosuppression orchestrated by cancer: towards an immuno-genomic paradigm for precision medicine. Nat. Rev. Clin. Oncol. *21*, 337–353. https://doi.org/10.1038/s41571-024-00870-6.
38. Kumagai, S., Togashi, Y., Kamada, T., Sugiyama, E., Nishinakamura, H., Takeuchi, Y., Vitaly, K., Itahashi, K., Maeda, Y., Matsui, S., et al. (2020). The PD-1 expression balance between effector and regulatory T cells predicts the clinical efficacy of PD-1 blockade therapies. Nat. Immunol. *21*, 1346–1358.
39. Italiano, A., Bessede, A., Pulido, M., Bompas, E., Piperno-Neumann, S., Chevreau, C., Penel, N., Bertucci, F., Toulmonde, M., Bellera, C., et al. (2022). Pembrolizumab in soft-tissue sarcomas with tertiary lymphoid structures: a phase 2 PEMBROSARC trial cohort. Nat. Med. *28*, 1199–1206.
40. Kinoshita, T., Ishii, G., Hiraoka, N., Hirayama, S., Yamauchi, C., Aokage, K., Hishida, T., Yoshida, J., Nagai, K., and Ochiai, A. (2013). Forkhead box P3 regulatory T cells coexisting with cancer associated fibroblasts are correlated with a poor outcome in lung adenocarcinoma. Cancer Sci. *104*, 409–415.
41. Karnoub, A.E., Dash, A.B., Vo, A.P., Sullivan, A., Brooks, M.W., Bell, G.W., Richardson, A.L., Polyak, K., Tubo, R., and Weinberg, R.A. (2007). Mesenchymal stem cells within tumour stroma promote breast cancer metastasis. Nature *449*, 557–563.
42. Tan, W., Zhang, W., Strasner, A., Grivennikov, S., Cheng, J.Q., Hoffman, R.M., and Karin, M. (2011). Tumour-infiltrating regulatory T cells stimulate mammary cancer metastasis through RANKL-RANK signalling. Nature *470*, 548–553.
43. Jacobs, J., Deschoolmeester, V., Zwaenepoel, K., Flieswasser, T., Deben, C., Van den Bossche, J., Hermans, C., Rolfo, C., Peeters, M., De Wever, O., et al. (2018). Unveiling a CD70-positive subset of cancer-associated fibroblasts marked by pro-migratory activity and thriving regulatory T cell accumulation. OncoImmunology *7*, e1440167.
44. Mao, X., Xu, J., Wang, W., Liang, C., Hua, J., Liu, J., Zhang, B., Meng, Q., Yu, X., and Shi, S. (2021). Crosstalk between cancer-associated fibroblasts and immune cells in the tumor microenvironment: new findings and future perspectives. Mol. Cancer *20*, 131.
45. Schwartz, L.H., Litière, S., de Vries, E., Ford, R., Gwyther, S., Mandrekar, S., Shankar, L., Bogaerts, J., Chen, A., Dancey, J., et al. (2016). RECIST 1.1 – Update and Clarification: From the RECIST Committee. Eur J Cancer Oxf Engl *62*, 132–137.
46. Vanhersecke, L., Bougouin, A., Crombé, A., Brunet, M., Sofeu, C., Parrens, M., Pierron, H., Bonhomme, B., Lembege, N., Rey, C., et al. (2023). Standardized Pathology Screening of Mature Tertiary Lymphoid Structures in Cancers. Lab. Invest. *103*, 100063.
47. Behr, D.S., Peitsch, W.K., Hametner, C., Lasitschka, F., Houben, R., Schönhaar, K., Michel, J., Dollt, C., Goebeler, M., Marx, A., et al. (2014).

CellPress
OPEN ACCESS

Prognostic value of immune cell infiltration, tertiary lymphoid structures and PD-L1 expression in Merkel cell carcinomas. Int. J. Clin. Exp. Pathol. *7*, 7610–7621.

48. Frampton, G.M., Fichtenholtz, A., Otto, G.A., Wang, K., Downing, S.R., He, J., Schnall-Levin, M., White, J., Sanford, E.M., An, P., et al. (2013). Development and validation of a clinical cancer genomic profiling test based on massively parallel DNA sequencing. Nat. Biotechnol. *31*, 1023–1031.

49. Zollinger, D.R., Lingle, S.E., Sorg, K., Beechem, J.M., and Merritt, C.R. (2020). GeoMx^{TM} RNA Assay: High Multiplex, Digital, Spatial Analysis of RNA in FFPE Tissue. Methods Mol. Biol. *2148*, 331–345.

50. Danaher, P., Kim, Y., Nelson, B., Griswold, M., Yang, Z., Piazza, E., and Beechem, J.M. (2022). Advances in mixed cell deconvolution enable quantification of cell types in spatial transcriptomic data. Nat. Commun. *13*, 385.

STAR★METHODS

KEY RESOURCES TABLE

REAGENT or RESOURCE	SOURCE	IDENTIFIER
Antibodies		
Anti-CD3 (2GV6) Rabbit Monoclonal Primary Antibody	Ventana/Roche	790–4341
Anti-CD20 (L26) Rabbit Monoclonal Primary Antibody	Ventana/Roche	760–2531
Anti-CD23 (SP23) Rabbit Monoclonal Primary Antibody	Ventana/Roche	790–4408
OmniMap anti-Rb HRP Rabbit	Ventana/Roche	760–4311
OMap anti-Ms HRP Mouse	Ventana/Roche	760–4310
Anti-PanCK (AE1/AE3) Mouse Monoclonal Primary Antibody	Novus	NBP2-33200AF532
Anti-CD45 (EM-05) Rat Monoclonal Primary Antibody	Novus	NBP1-44763AF594
SYTO Green Fluorescent Nucleic Acid Stains	Invitrogen	10413072
Anti-α-SMA (D4K9N) Rabbit Monoclonal Primary Antibody	CST	19245
Anti-FAP (EPR20021) Rabbit Monoclonal Primary Antibody	ABCAM	ab207178
Anti-MYH11 (EPR5336(B)) Rabbit Monoclonal Primary Antibody	ABCAM	ab133567
Anti-CD8 (C8/144B) Mouse Monoclonal Primary Antibody	Dako	M7103
Anti-PanCK (AE1/AE3/PCK26) Mouse Monoclonal Primary Antibody	Ventana/Roche	760–2595
Anti-PD1 (NAT105) Mouse Monoclonal Primary Antibody	Roche	760–4985
Anti-TIM3 (D5D5R) Rabbit Monoclonal Primary Antibody	CST	45208
Anti-LAG3 (EP294) Rabbit Monoclonal Primary Antibody	EPITHOMICS	BSB3367
Anti-TIGIT (BLR047F) Rabbit Monoclonal Primary Antibody	ABCAM	AB243903
Anti-CD39 (EPR20627) Rabbit Monoclonal Primary Antibody	ABCAM	ab223842
Anti-CD4 (SP35) Rabbit Monoclonal Primary Antibody	Ventana/Roche	790–4423
Anti-FOXP3 (236A/E7) Mouse Monoclonal Primary Antibody	ABCAM	ab20034
Anti-ICOS (D1K2T) Rabbit Monoclonal Primary Antibody	CST	89601
Opal fluorophores	Akoya Biosciences	https://www.akoyabio.com/phenoimager/assays/opal-fluorophore-reagent-packs/
Biological samples		
FFPE human tumor samples	Institut Bergonié Department of Pathology	https://www.bergonie.fr/
Critical commercial assays		
Illumina i5 and i7 dual-indexing primers	Illumina	https://emea.illumina.com/products/by-type/sequencing-kits/library-prep-kits/stranded-mrna-prep.html

(Continued on next page)

Continued

REAGENT or RESOURCE	SOURCE	IDENTIFIER
NextSeq 2000 system	Illumina	https://www.illumina.com/systems/sequencing-platforms/nextseq-1000-2000.html
Opal Fluorophore Reagent Packs	Akoya Biosciences	https://www.akoyabio.com/phenoimager/assays/opal-fluorophore-reagent-packs/
Deposited data		
RNAseq signatures of T cells exhaustion pathway	This paper, Kim data, Wu data, Guo data, Zheng data, Hsu data, Zhang data, Egelston data, Niwon data, Kuang data	https://doi.org/10.1038/s41467-020-16164-1, https://doi.org/10.1038/s41598-023-40662-z, https://doi.org/10.1038/s41591-018-0045-3, https://doi.org/10.1016/j.cell.2017.05.035, https://doi.org/10.1159/000515305, https://doi.org/10.1016/j.ebiom.2022.104207, https://10.1172/jci.insight.153963, https://doi.org/10.1016/j.immuni.2022.10.002, https://doi.org/10.18632/aging.204830,
RNAseq signatures of regulatory T cells pathway	This paper, Schroeder data, Szabo data, Devi-Marulkar data, Freeman data, DeSimone data, Angelova data	https://doi.org/10.3390/cancers14051290, https://doi.org/10.1038/s41467-019-12464-3, https://doi.org/10.1038/s42003-022-04356-y, https://doi.org/10.1172/jci128672, https://doi.org/10.1016/j.immuni.2016.10.021, https://doi.org/10.1186/s13059-015-0620-6
Oligonucleotides		
Oligonucleotide probe mix (human WTA)	Nanostring	https://nanostring.com/wp-content/uploads/PB_MK3683_GeoMx-WTA_r9.pdf
Software and algorithms		
limma R package	Bioconductor	https://www.bioconductor.org/packages/release/bioc/html/limma.html
SpatialDecon R package	Bioconductor	https://bioconductor.org/packages/release/bioc/html/SpatialDecon.html
inForm v.2.60	Akoya Biosciences	https://www.akoyabio.com/phenoimager/inform-tissue-finder/
GaussNorm function from the flowStats R package	Bioconductor	https://www.bioconductor.org/packages/release/bioc/html/flowStats.html
FlowJo software (v.10.8.0)	FlowJo	https://www.flowjo.com/solutions/flowjo/downloads/previous-versions
phenoptrReports R package	Akoya	https://www.akoyabio.com/wp-content/uploads/2021/12/PhenoptrRerports_Install_Instructions.v5.pdf
fgsea R package	Bioconductor	https://bioconductor.org/packages/release/bioc/html/fgsea.html
EnhancedVolcano R package	Bioconductor	https://bioconductor.org/packages/release/bioc/html/EnhancedVolcano.html
survival R package	CRAN	https://cran.r-project.org/web/packages/survival/index.html
survminer R package	CRAN	https://cran.r-project.org/web/packages/survminer/index.html
survivalAnalysis R package	CRAN	https://cran.r-project.org/web/packages/survivalAnalysis/index.html
Other		
Bergonié Institute Profiling precision medicine study	N/A	NCT02534649
Ventana Discovery Ultra platform	Ventana; Roche Diagnostics	https://diagnostics.roche.com/us/en/products/product-category/immunohistochemistry–ihc-/discovery-ultra.html

(*Continued on next page*)

Continued		
REAGENT or RESOURCE	SOURCE	IDENTIFIER
Phenoimager^{HT} multispectral imaging system	Akoya Biosciences	https://www.akoyabio.com/phenoimager/instruments/phenoimager-ht/
FoundationOne® CDx NGS	Roche	https://www.rochefoundationmedicine.com/home/services.html
GeoMx® DSP Whole Transcriptome Atlas	Nanostring	https://nanostring.com/products/geomx-digital-spatial-profiler/geomx-dsp-overview/
Ventana Discovery XT platform	Ventana; Roche Diagnostics	https://diagnostics.roche.com/fr/fr/products/tests/discovery-research-reagents.html
Phenochart slide viewer	Akoya	https://www.akoyabio.com/support/software/
HTG Transcriptome Panel (HTP)	HTG Molecular Diagnostics	https://www.htgmolecular.com/assays/htp
RStudio software	RStudio, Inc.	https://github.com/rstudio/rstudio

EXPERIMENTAL MODEL AND STUDY PARTICIPANT DETAILS

Patient population

This study drawed on data from individuals enrolled in the BIP precision medicine study (NCT02534649, https://clinicaltrials.gov/study/NCT02534649) led by Institut Bergonié, Bordeaux, France, spanning from December 2015 to December 2023 (Table S1). Participants included those over 18 years of age, diagnosed with histologically confirmed non-small cell lung cancer (NSCLC), exhibiting unresectable and/or metastatic disease. Eligibility required at least one tumor evaluation post-initiation of an immune checkpoint inhibitor-based regimen and access to pre-immunotherapy formalin-fixed paraffin-embedded (FFPE) tumor samples. The study received approval from the institutional ethics review board, with patients providing informed consent. A total of 509 patients were included. No influence of sex or gender was observed.

Treatments and evaluation

Treatment protocols were determined by the attending physicians, with tumor evaluations scheduled every 6–8 weeks as per the standard care practice. The evaluation of treatment responses adhered to RECIST1.1 criteria, with responders (R) defined as achieving either complete response (CR) or partial response (PR), and non-responders (NR) defined as achieving either stable disease (SD) or progressive disease (PD).[45] Progression-free survival (PFS) and overall survival (OS) were measured from the initiation of treatment to the occurrence of disease progression, patient death, or the last patient contact, respectively.

METHOD DETAILS

Immunohistochemistry (IHC) stainings and TLS assessment

All stainings were conducted on 3.5-μm thick FFPE slides using the Ventana Discovery Ultra platform (Ventana; Roche Diagnostics). Multiplexed immunohistochemistry (mIHC) staining involved CD3 (2GV6; Ventana), CD20 (L26; Ventana) and CD23 (clone SP12; Ventana) antibodies. The staining protocol, RUO discovery universal, was followed according to the manufacturer's instructions, utilizing OmniMap anti-Rb HRP (760–4311; Ventana) and OmniMap anti-Ms HRP (760–4310; Ventana) detection kits. The Phenoimager^{HT} multispectral imaging system (Akoya Biosciences) was used for slides digitalization.

Expert pathologists (L.V., J.M.C) reviewed all samples to assess the presence and maturity of TLS based on hematoxylin and eosin (H&E) and mIHC staining on consecutive sections, consistent with the current standardized pathology definition.[46] Briefly, TLS were defined as clusters of lymphoid aggregates of at least 50 CD20^{+} B cells, admixed with a variable proportion of CD3^{+} T cells, and located either among the tumor cells or at the invasive margin (defined as a fibrous tissue distance of <1 mm from tumor cells), as previously described.[14,46,47] When the TLS status was assessed on lymphoid organs (namely, the lymph nodes, spleen and tonsils), TLSs were only taken into account when admixed with tumor cells and if distant from the residual parenchyma, to exclude pre-existing lymphoid follicles. TLSs were classified as mature when at least one CD23^{+} dendritic cell was detected within them. When isolated CD23-positive cells were detected, they had to display a dendritic morphology (that is, cytoplasmic dendritic extensions) to be considered significant. In the absence of CD23 positivity, TLSs were defined as immature. Of note, mTLSs displaying prominent germinal centers on HES staining were systematically confirmed with CD23 staining. The study analyzed both biopsies and surgical specimens. A total of 77 patients were included.

Genomic, tumor mutation burden (TMB) and microsatellite instability (MSI) analysis

Tumor genomic profiling analysis was conducted using the FDA-approved FoundationOne CDx, which includes the analysis of a 324 genes panel. This comprehensive analysis encompasses the calculation of tumor mutational burden (TMB) and microsatellite instability (MSI).[48]

Spatial transcriptomics

Spatial transcriptomic data were acquired as described previously.[39] Briefly, a total of six patients with available surgical FFPE specimens were selected based on their response to immunotherapy: three patients with objective responses (responder, R) and three patients with progressive disease (non-responder, NR; Table S2). For the analysis, 5μm FFPE slides were prepared for the NanoString Whole Transcriptome Atlas according to NanoString's instructions. After paraffin removal by baking the slides for 2 h, they were rehydrated, subjected to heat-induced epitope retrieval (20 min at 100°C), and enzymatic digestion (0.1 μg mL−1 proteinase K for 10 min at 37°C). The tissue sections were hybridized with the oligonucleotide probe mix (human WTA) overnight, followed by two 5-min washes, blocking, and incubation with morphology marker antibodies: PanCK (AE1+AE3; 532 channel; Novus) and CD45 (EM-05; 594 channel; Novus), with Syto13 (488 channels; Invitrogen) used as a nuclear stain. The tissue sections were loaded into the GeoMx Digital Spatial Profiling platform, and regions of interest (ROIs) on TLS and tumor areas were selected. The TLS were previously identified on serial FFPE section using a multiplex IHF panel illustrated in Figures S3A and S3B and selected for the GeoMx experiments. These ROIs, chosen as representative parts of the TME, were further segmented into $CD45^+$ and $CD45^-$ areas for TLSs in order to differentiate between immune cells and other stromal cells, as well as into $PanCK^+/CD45^-$ and $PanCK^-/CD45^{+/-}$ areas for tumor regions to separate tumor cells from non-tumor cells (Table S3). Ultraviolet light was used to release and collect RNA ID and unique molecular identifier-containing oligonucleotide tags, with Illumina i5 and i7 dual-indexing primers added during PCR to uniquely index each AOI. After PCR purification, library concentration and quality were assessed before sequencing on a NextSeq 2000 system. Fastq files were processed, resulting in count data for each probe in each AOI. Quality control and principal component analysis were performed to ensure data integrity, with raw counts normalized using full quantile normalization. Differentially expressed genes were analyzed using the limma R package (https://bioconductor.org/packages/release/bioc/html/limma.html), employing patient ID as a blocking factor to account for variation in the number of AOIs analyzed per patient. The distribution of AOIs was balanced between R and NR, with no need for statistical adjustment based on the number of AOIs available per patient[49] (Table S3). Cell type estimation was conducted using the SpatialDecon algorithm developed by NanoString.[50] The differences in fibroblast cell abundances between R and NR were tested in the $PanCK^-/CD45^-$ regions (Figures S3C–S3G).

Multiplex immunohistofluorescence assay

To set up the multiplex immunohistofluorescence (mIF) panel, the staining conditions for each antibody were initially optimized through IHC, with a pathologist validating the staining patterns (J.M.C.). The positioning of each antibody within the mIF panel was determined according to signal resistance through several stripping cycles. Each antibody was then paired with a specific Opal fluorochrome tailored to the target expression level and the antibody's position within the panel to minimize signal spillover. The validation of the mIF panel was finally determined by comparing signals from monoplex staining with mIF staining.

Multiplex immunohistofluorescence was carried out on the Ventana Discovery XT staining platform (Ventana). The process started with the deparaffinization of tumor tissue slides followed by antigen retrieval using the standard CC1 reagent. Slides were then incubated with primary antibodies targeting the following molecules, as per the panel composition: αSMA (D4K9N, CST), MYH11 (EPR5336(B), Abcam), FAP (EPR20021, Abcam), CD8 (C8/144B, Abcam), and PanCK (AE1/AE3/PCK26, Ventana) for the fibroblast panel; PD1 (NAT105, Cell Marque), TIM3 (D5D5R, Cell Signaling Technology), LAG3 (EP294, BioSB), CD8 (C8/144B, Dako), TIGIT (BLR047F, Abcam), CD39 (EPR20627, Abcam) for the exhaustion panel; and CD4 (SP35, Ventana), CD8 (C8/144B, Dako), CD20 (L26, Ventana), FoxP3 (236A/E7, Abcam), ICOS (D1K2T, CST), and TIGIT (BLR047F, Abcam) for the regulatory T cells panel. Detection of bound primary antibodies was conducted using OmniMap HRP-conjugated anti-rabbit IgG (760–4311; Ventana; Roche) and OmniMap HRP-conjugated anti-mouse IgG (760–4310; Ventana; Roche), followed by tyramide signal amplification using Opal fluorophores (Opal 480, Opal 520, Opal 570, Opal 620, Opal 690, and Opal 780; Akoya Biosciences). Finally, the slides were counterstained with spectral 4′,6-diamidino-2-phenylindole (DAPI; Akoya Biosciences), cover-slipped, and digitized using the multispectral imaging platform PhenoImager HT (Akoya).

The images acquired through multispectral imaging were unmixed using spectral libraries built from images stained for each fluorophore (monoplex) and analyzed with the inForm Advanced Image Analysis software (inForm v.2.6.0; Akoya Biosciences). A trained pathologist (L.V., J.M.C) delineated tumor areas on each tissue slide using PhenoChart (Akoya Biosciences), and the annotated sections were analyzed with inForm software (v.2.6.0). Tissues were segmented into "tumor" versus "stroma" areas based on PanCK staining, with cell segmentation guided by DAPI and fluorescent membrane signals. Mean fluorescent marker intensities for each cell were extracted, and signal intensities were normalized using the GaussNorm function from the flowStats R package (v4.8.2). Cells were then phenotyped employing a thresholding method in FlowJo software (v.10.8.0; FlowJo), and intercellular distances between $PanCK^+$ and $CD8^+$ cells were calculated using the phenoptrReports (v0.3.2) R package.

Quantitative analysis of T cells infiltrate (immune contexture)

The density of $CD8^+$ T cells in each sample was semi-automatically quantified using the Inform software (Akoya Biosciences; version 2.6.0), following tissue segmentation and digital cell phenotyping. $CD8^+$ T cell distribution patterns within the TME are typically classified into three categories: infiltrated (or inflamed), excluded, and desert.[12] The infiltrated profile features $CD8^+$ T cells within the tumor parenchyma. The excluded profile is identified by the presence of $CD8^+$ T cells in the stroma surrounding tumor cell nests without penetrating the parenchyma. This stromal presence can be peripheral, limited to the tumor capsule, or diffuse, penetrating the tumor itself. The desert profile is defined by a significant lack or complete absence of $CD8^+$ T cells in both the parenchyma and the stroma of

the tumor. An optimal cutpoint of 5% is used to differentiate between the desert profile and others, as well as to distinguish the excluded profile from the infiltrated one.

Regional HTG transcriptomic analysis

Regional transcriptomic analysis was conducted on FFPE samples using the quantitative nuclease protection assay of the HTG Transcriptome Panel (HTP), according to the manufacturer's protocol from HTG Molecular Diagnostics (Tucson, AZ, USA). The HTP comprises 19,616 Nuclease Protection Probes (NPPs), including 19,398 target probes, 100 negative control probes, 92 probes for RNA controls as established by the external RNA control consortium (ERCC) probes, 22 probes for gDNA measurement, and 4 positive control (POS) probes. Initially, tissue regions were macrodissected from 5-μm thick FFPE sections, followed by lysis and Proteinase K treatment. Post-protein inactivation, DNase digestion was performed, and the quantitative nuclease protection assay was run using HTG EdgeSeq Processors. Adapters and sample tags were then added during PCR amplification, and the sequenced libraries on an Illumina NextSeq 2000 resulted in FASTQ files. These files were processed into a gene expression count matrix using HTG EdgeSeq Reveal Software.

For the enrichment analyses of hallmark terms, exhaustion signatures, and regulatory T cell signatures, the fgsea R package (v1.28.0) was utilized with specified gene signatures (Tables S6 and S7). Visualization of the data, including volcano plots and heatmaps, was achieved using the EnhancedVolcano (v1.20.0) and pheatmap (v1.0.12) packages, respectively.

QUANTIFICATION AND STATISTICAL ANALYSIS

The cut-off date for statistical analysis of baseline demographic data and clinical outcomes was set for December 31, 2023. To understand the distribution of variables within the study population, descriptive statistics were employed. Group differences were assessed using chi-squared tests or Fisher's exact tests for categorical variables, and Student's t-tests were applied to continuous variables. For survival analysis, Kaplan-Meier survival curves were compared using the log rank test, facilitated by the survival R package (version 3.3.1). The Cox proportional hazards regression model helped estimate hazard ratios (HR) and 95% confidence intervals (CI), categorizing patients into CAF-High or CAF-Low groups, and CAF-High, CAF-Mid or CAF-Low groups, respectively. This categorization was based on a threshold optimized through maximally selected rank statistics, using progression-free survival (PFS) as the outcome measure (survminer R package, version 0.4.9).

Furthermore, multivariable Cox proportional hazards regression models examined the interdependence of various biomarkers to predict the benefit from ICIs, utilizing the survivalAnalysis R package (version 0.3.0). All statistical tests were conducted bidirectionally, with a *p*-value of less than 0.05 denoting statistical significance. These analyses were carried out using R software (version 4.2.1).

The identification of prognostic factors involved both univariate and multivariate analyses through a Cox regression model. The multivariate analysis considered several variables, including age, sex, performance status, the number of previous treatment lines, histologic subtype, treatment regimen, PD-L1 expression status (as determined by TPS), and TLS status, using the survivalAnalysis package in R (version 0.3.0) for the computations.

ADDITIONAL RESOURCES

This study drawed on data from individuals enrolled in the BIP precision medicine study (NCT02534649, https://clinicaltrials.gov/study/NCT02534649) led by Institut Bergonié, Bordeaux, France, spanning from December 2015 to December 2023.

Cell Reports Medicine, Volume *6*

Supplemental information

Spatially resolved transcriptomics reveal the determinants of primary resistance to immunotherapy in NSCLC with mature tertiary lymphoid structures

Florent Peyraud, Jean-Philippe Guégan, Christophe Rey, Oren Lara, Ophélie Odin, Marie Del Castillo, Lucile Vanhersecke, Jean-Michel Coindre, Emma Clot, Maxime Brunet, Thomas Grellety, Angélique Tasseel, Sylvestre Le Moulec, Robert J. Johnston, Alban Bessede, and Antoine Italiano

SUPPLEMENTAL INFORMATION

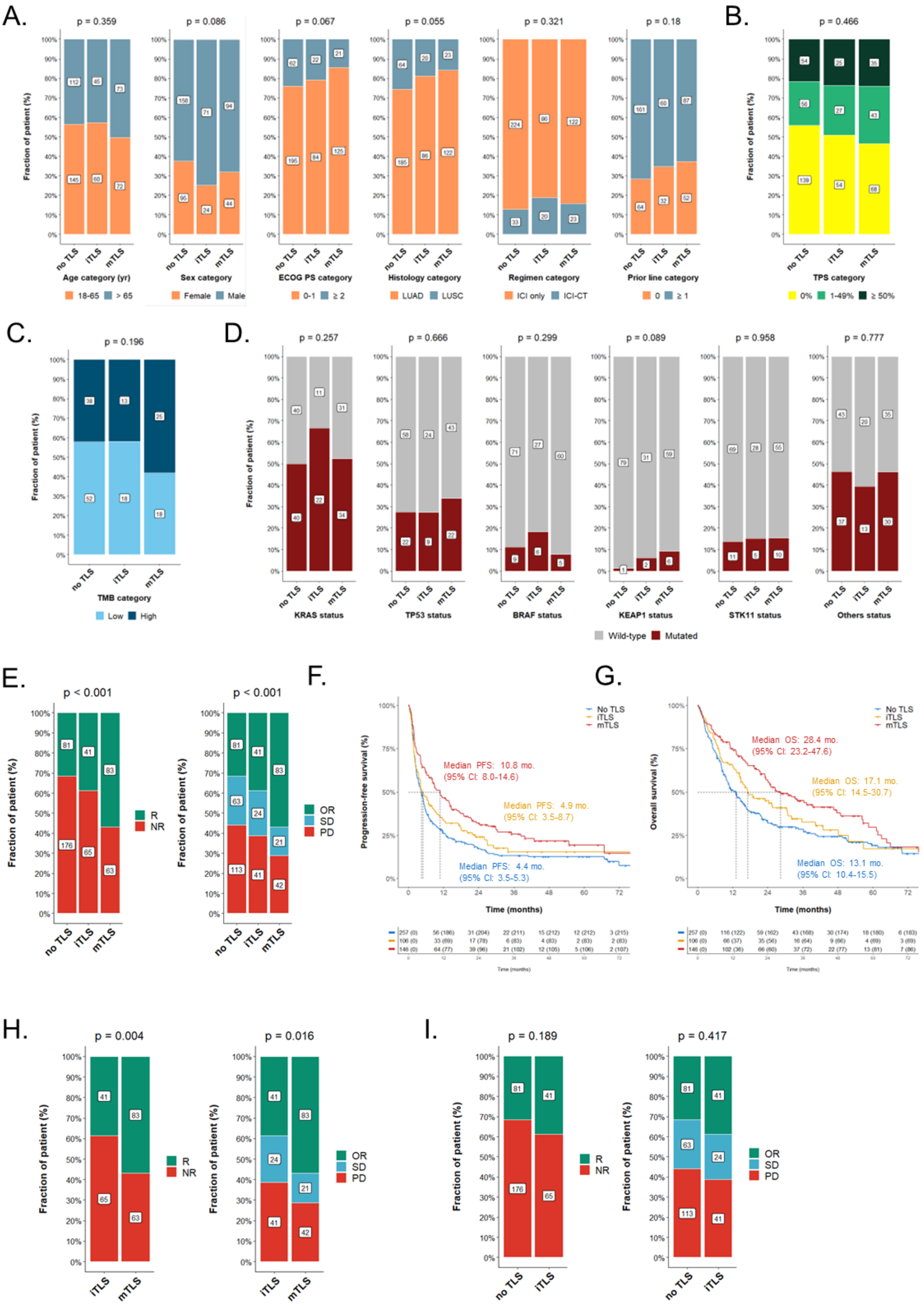

Figure S1. Characterization of ICI-treated NSCLC patients based on TLS status. Related to Figure 1.

A. Distribution of baseline characteristics of patients according to the TLS status. Statistical significance was determined by chi-squared test.

B. Distribution of PD-L1 tumor expression (TPS) of NSCLC samples according to the TLS status. Statistical significance was determined by chi-squared test.

C. Distribution of tumor mutational burden (TMB) of NSCLC samples according to the TLS status. Statistical significance was determined by chi-squared test.

D. Distribution of mutational status of NSCLC samples according to the TLS status. Statistical significance was determined by chi-squared test.

E. Response rate, as defined per objective response (left) or RECIST 1.1 criteria (right), according to TLS status: absence (negative, no TLS) , iTLS or mTLS. Statistical significance was determined by chi-squared test.

F. Kaplan–Meier analysis of the PFS of patients according to TLS status (n=509; red curve: mature TLS-enriched tumors; orange curve: immature TLS-enriched tumors; blue curve: TLS-negative tumors). Numbers below each x axis indicate the number of patients at risk and those in parentheses are the number of events.

G. Kaplan–Meier analysis of the OS of patients according to TLS status (n=509; red curve: mature TLS-enriched tumors; orange curve: immature TLS-enriched tumors; blue curve: TLS-negative tumors). Numbers below each x axis indicate the number of patients at risk and those in parentheses are the number of events.

H. Response rate, as defined per objective response (left) or RECIST 1.1 criteria (right), according to TLS status: immature TLS (iTLS) or mature TLS (mTLS). Statistical significance was determined by chi-squared test.

I. Response rate, as defined per objective response (left) or RECIST 1.1 criteria (right), according to TLS status: absence (no TLS) or immature TLS (iTLS). Statistical significance was determined by chi-squared test.

CI: confidence interval; ECOG PS: Eastern Cooperative Oncology Group Performance Status; ICI: immune checkpoint inhibitors; iTLS: immature TLS; mTLS: mature TLS; no TLS: absence of TLS; OR, objective response; PD, progressive disease; SD, stable disease; R, responder; NR, non-responder.

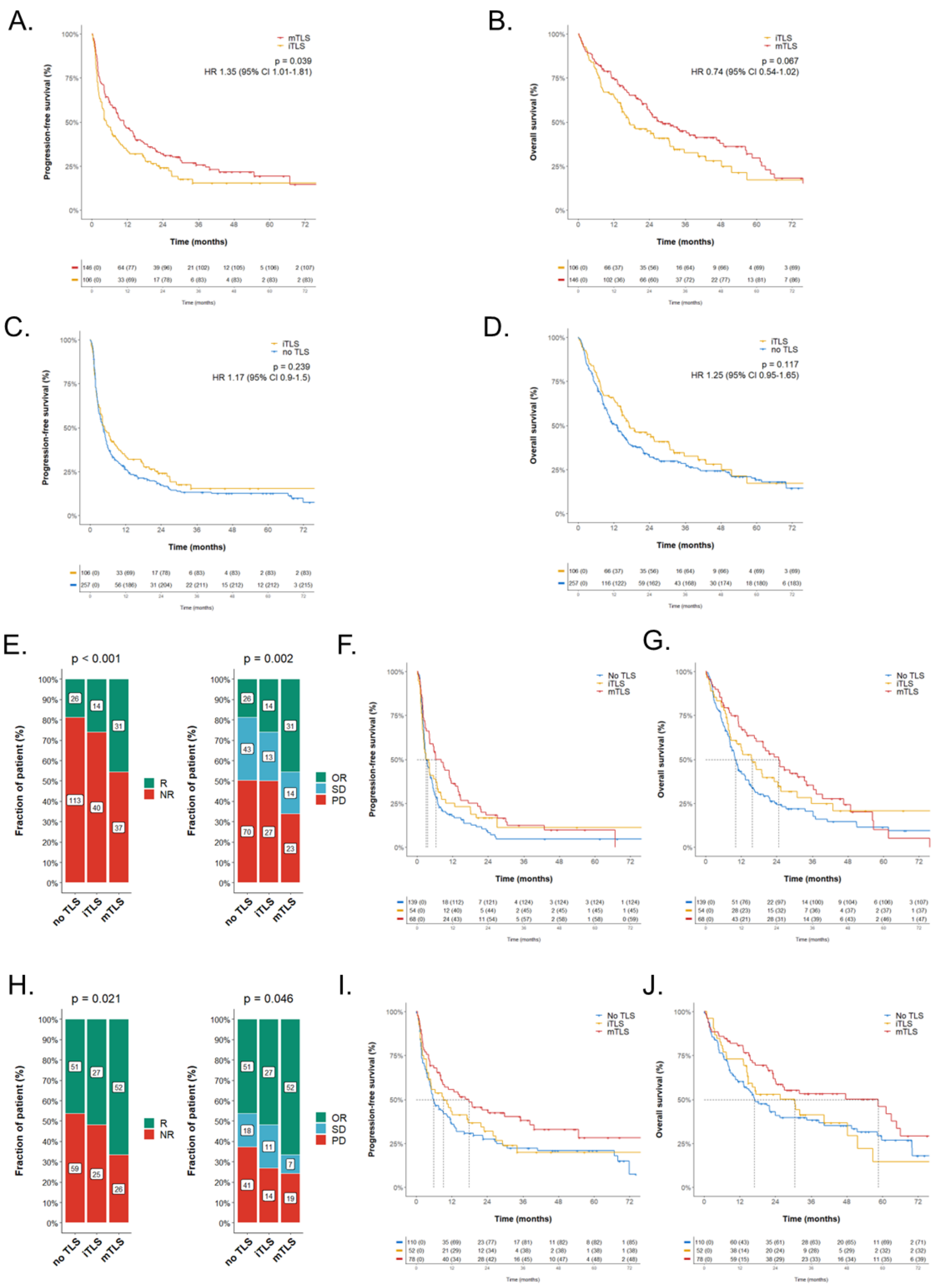

Figure S2. Clinical outcomes of ICI-treated NSCLC patients based on TLS status. Related to Figure 1.

A. Kaplan–Meier curves of the PFS of patients according to TLS maturity status (red curve: mTLS-enriched tumors; orange curve: iTLS-enriched tumors). Numbers below each x axis indicate the number of patients at risk and those in parentheses are the number of events. Statistical significance was determined by log-rank test.

B. Kaplan–Meier curves of the OS of patients according to TLS maturity status (red curve: mTLS-enriched tumors; orange curve: iTLS-enriched tumors). Numbers below each x axis indicate the number of patients at risk and those in parentheses are the number of events. Statistical significance was determined by log-rank test.

C. Kaplan–Meier curves of the PFS of patients according to TLS maturity status (blue curve: TLS-negative tumors; orange curve: iTLS-enriched tumors). Numbers below each x axis indicate the number of patients at risk and those in parentheses are the number of events. Statistical significance was determined by log-rank test.

D. Kaplan–Meier curves of the OS of patients according to TLS maturity status (blue curve: TLS-negative tumors; orange curve: iTLS-enriched tumors). Numbers below each x axis indicate the number of patients at risk and those in parentheses are the number of events. Statistical significance was determined by log-rank test.

E. Response rate, as defined per objective response (left) or RECIST 1.1 criteria (right), according to TLS status in PD-L1 negative tumors. Statistical significance was determined by chi-squared test.

F. Kaplan–Meier curves of the PFS of patients according to TLS maturity status in PD-L1 negative tumors (red curve: mTLS-enriched tumors; orange curve: iTLS-enriched tumors; blue curve: TLS-negative tumors). Numbers below each x axis indicate the number of patients at risk and those in parentheses are the number of events.

G. Kaplan–Meier curves of the OS of patients according to TLS maturity status in PD-L1 negative tumors (red curve: mTLS-enriched tumors; orange curve: iTLS-enriched tumors; blue curve: TLS-negative tumors). Numbers below each x axis indicate the number of patients at risk and those in parentheses are the number of events.

H. Response rate, as defined per objective response (left) or RECIST 1.1 criteria (right), according to TLS status in PD-L1 positive tumors. Statistical significance was determined by chi-squared test.

I. Kaplan–Meier curves of the PFS of patients according to TLS maturity status in PD-L1 positive tumors (red curve: mTLS-enriched tumors; orange curve: iTLS-enriched tumors; blue curve: TLS-negative tumors). Numbers below each x axis indicate the number of patients at risk and those in parentheses are the number of events.

J. Kaplan–Meier curves of the OS of patients according to TLS maturity status in PD-L1 positive tumors (red curve: mTLS-enriched tumors; orange curve: iTLS-enriched tumors; blue curve:

TLS-negative tumors). Numbers below each x axis indicate the number of patients at risk and those in parentheses are the number of events.

ECOG PS: Eastern Cooperative Oncology Group Performance Status; ICI: immune checkpoint inhibitors; iTLS: immature TLS; mTLS: mature TLS; no TLS: absence of TLS; OR, objective response; PD, progressive disease; SD, stable disease; R, responder; NR, non-responder.

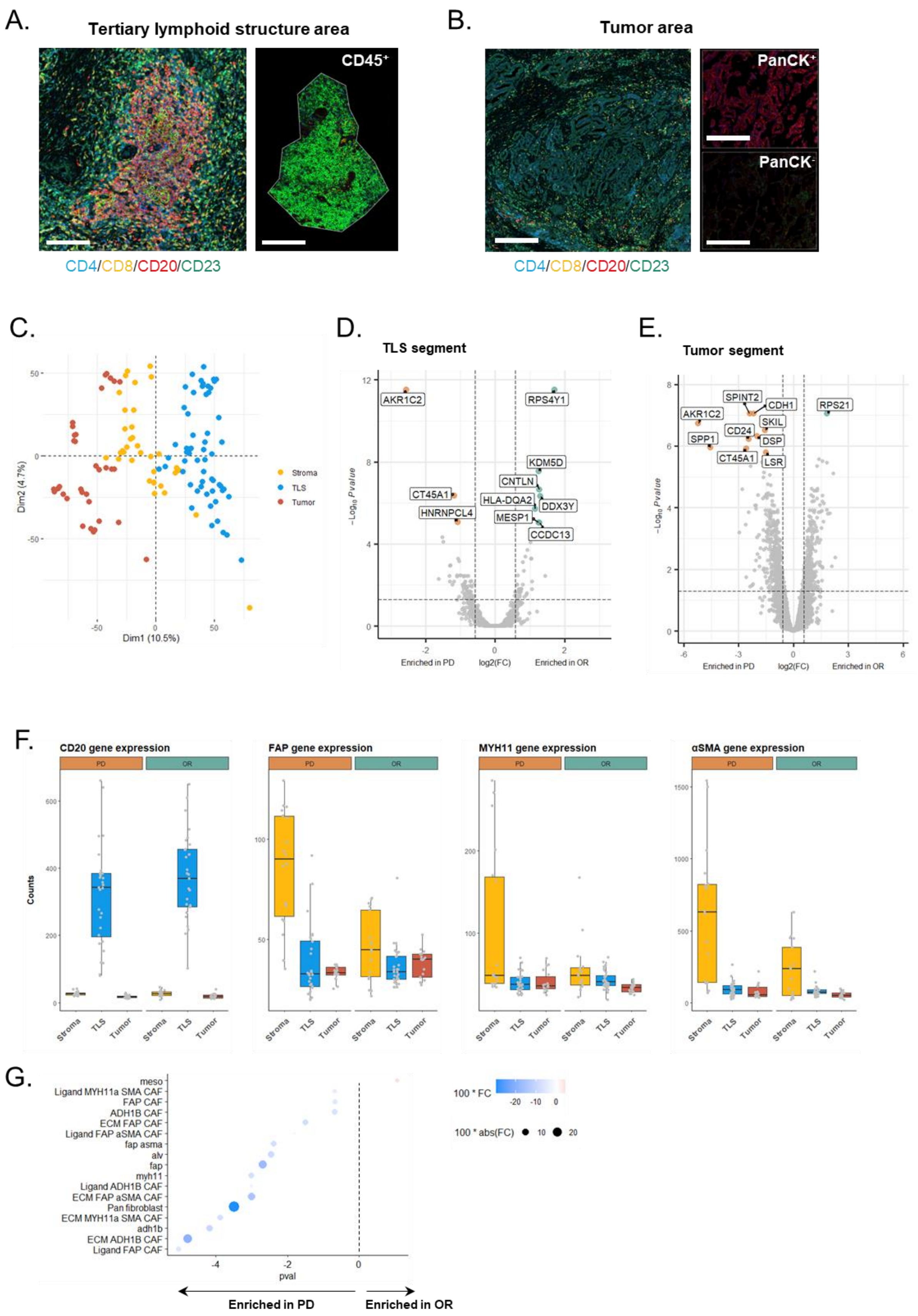

Figure S3. Spatial transcriptomic analysis of ICI-treated TLS-positive NSCLC. Related to Figure 2.

A. Representative illustration of a GeoMx DSP-analyzed TLS area. CD4/CD8/CD20/CD23 multiplex IHF staining (left) and CD45 (right) segmentation mask for GeoMx transcriptomics analysis. All scale bars, 50µm.

B. Representative illustration of a GeoMx DSP-analyzed tumor area. CD4/CD8/CD20/CD23 multiplex IHF staining (left) and PanCK (right) segmentation masks (righ panel, positive cells [top] and negative cells [bottom]) for GeoMx transcriptomics analysis. All scale bars, 200µm.

C. Principal component analysis (PCA) plot displaying AOIs according to segment.

D. Volcano plot of differentially genes expression between responders (PD, N=3) and non responders (OR, N=3) in TLS segment. Top 10 upregulated genes are displayed.

E. Volcano plot of differentially genes expression between responders (PD, N=3) and non responders (OR, N=3) in tumor segment. Top 10 upregulated genes are displayed.

F. Gene expression of CD20, FAP, MYH11 and aSMA in TLS, tumor and stroma segments, respectively, between non responders (PD, N=3) and responders (OR, N=3). Data are represented as median +/- IQR.

G. Bubble plot of publicly available scRNAseq gene signature from Grout et al. (TCGA and external dataset) estimated with deconvolution algorithm between non responders (PD, N=3) and responders (OR, N=3) or the GeoMx spatial transcriptomic experiment.

FC: fold change; IQR: interquartile range; TLS: tertiary lymphoid structure.

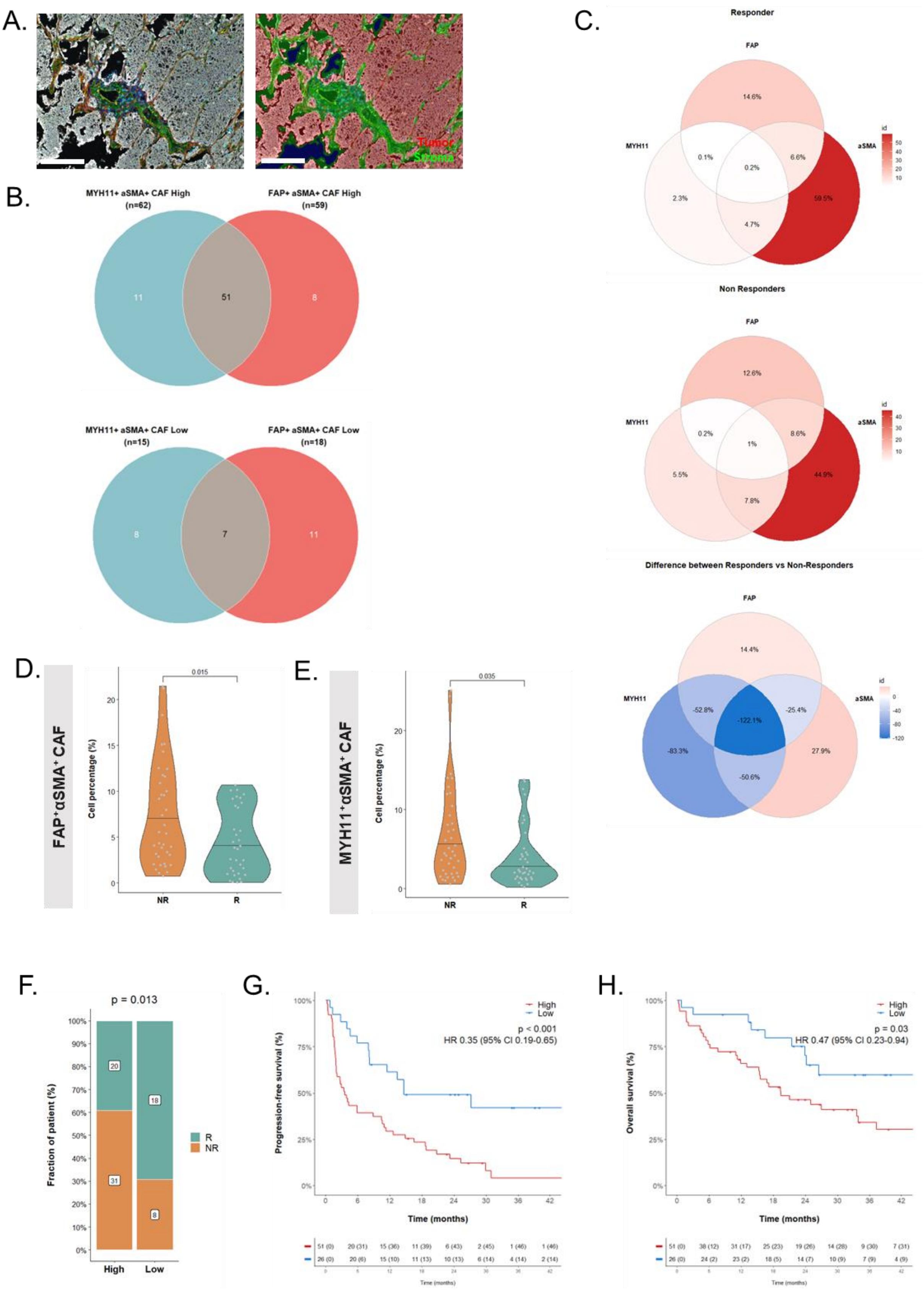

Figure S4. Clinical outcomes of ICI-treated TLS-positive NSCLC patients based on CAFs status. Related to Figure 3.

A. Illustration of the segmentation strategy of the tissue in “Stroma” and “Tumor” areas. All scale bars, 200µm.

B. Venn diagram depicting individuals CAF category with MYH11$^+$αSMA$^+$ CAF and/or FAP$^+$αSMA$^+$ CAF High (upper) and with MYH11$^+$αSMA$^+$ CAF and/or FAP$^+$αSMA$^+$ CAF Low (bottom).

C. Venn diagram of CAF marker expression of NSCLC Responders (upper - R) and Non-Responders (middle - NR). Differences in cells proportion between responders and non-responders are displayed on bottom. For the analysis, cohort (n=77 patients) was down-sampled to 5000 cells per patient. Median of cell distribution is shown.

D. Percentage of FAP$^+$αSMA$^+$ CAF in the stroma areas of responders and non-responders to ICI. The P values were calculated using Wilcoxon tests. Data are represented as median.

E. Percentage of MYH11$^+$αSMA$^+$ CAF in the stroma areas of responders and non-responders to ICI. The P values were calculated using Wilcoxon tests. Data are represented as median.

F. Proportion of patients with high and low density of combined FAP$^+$αSMA$^+$ and MYH11$^+$αSMA$^+$ CAF according to response. The P value was calculated using a X2 test.

G. Kaplan–Meier curves of the PFS of patients classified as high or low based on levels of combined FAP$^+$αSMA$^+$ and MYH11$^+$αSMA$^+$ CAF .

H. Kaplan–Meier curves of the OS of patients classified as high or low based on levels of combined FAP$^+$αSMA$^+$ and MYH11$^+$αSMA$^+$ CAF .

CAF: cancer-associated fibroblast; ICI: immune checkpoint inhibitor; NSCLC: non-small cell lung cancer.

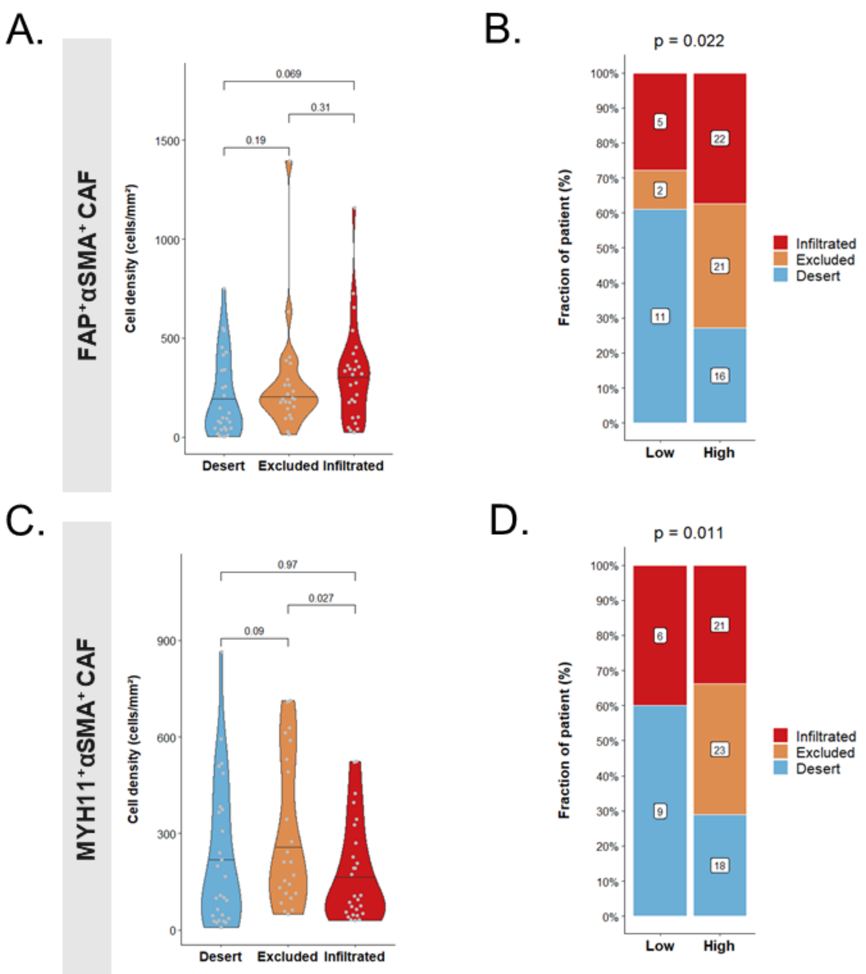

Figure S5. Analysis of CAF density and category based on immune contexture. Related to Figure 3.

A. Density of FAP⁺αSMA⁺ CAF in the stroma according to immune contexture. The P values were calculated using Wilcoxon tests. Data are represented as median.
B. Proportion of patients with infiltrated, excluded and desert immune contexture according to density of FAP⁺αSMA⁺ CAF category. The P value was calculated using chi-squared test.
C. Density of MYH11⁺αSMA⁺ CAF in the stroma according to immune contexture. The P values were calculated using Wilcoxon tests. Data are represented as median.
D. Proportion of patients with infiltrated, excluded and desert immune contexture according to density of MYH11⁺αSMA⁺ CAF category. The P value was calculated using chi-squared test.

CAF: cancer-associated fibroblast.

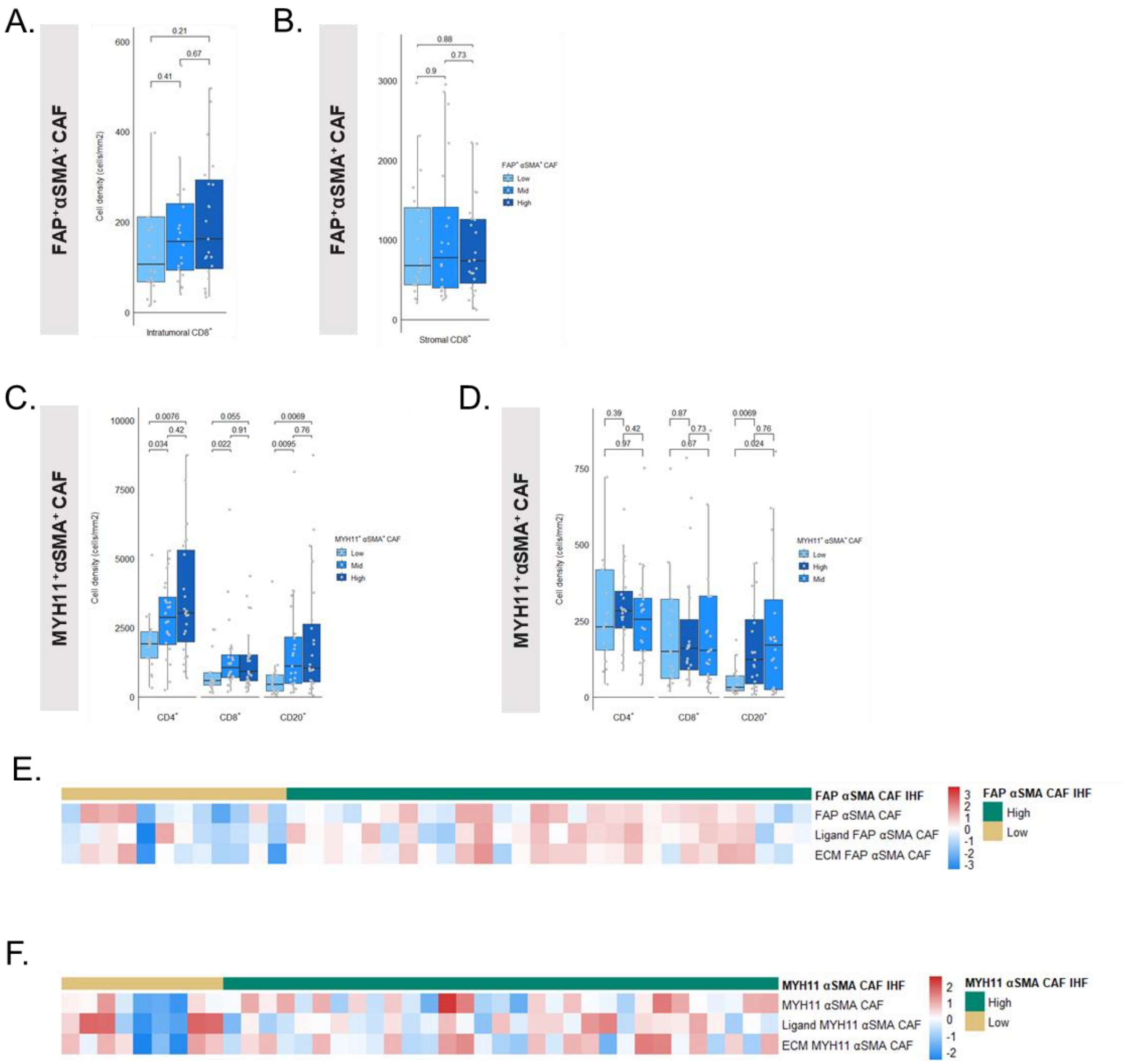

Figure S6. Analysis of T and B cells infiltrate based on CAF subsets. Related to Figure 4 and 5.

A. Density of intratumoral CD8⁺ T cells according to stromal FAP⁺αSMA⁺ CAF category. The P values were calculated using Wilcoxon tests. Data are represented as median +/- IQR.

B. Density of stromal CD8⁺ T cells according to stromal FAP⁺αSMA⁺ CAF category. The P values were calculated using Wilcoxon tests. Data are represented as median +/- IQR.

C. Density of stromal CD4⁺, CD8⁺ and CD20⁺ T cells according to stromal MYH11⁺αSMA⁺ CAF category. The P values were calculated using Wilcoxon tests. Data are represented as median +/- IQR.

D. Density of intratumoral CD4⁺, CD8⁺ and CD20⁺ T cells according to stromal MYH11⁺αSMA⁺ CAF category. The P values were calculated using Wilcoxon tests. Data are represented as median +/- IQR.

E. Heatmap of scRNAseq FAP^{+}αSMA^{+} CAF, its ligand or its ECM signatures, respectively, according to FAP^{+}αSMA^{+} CAF IHF category. FAP^{+}αSMA^{+} CAF phenotype is identified by IHF on the matched FFPE samples and then used to stratify samples.

F. Heatmap of scRNAseq MYH11^{+}αSMA^{+} CAF, its ligand or its ECM signatures, respectively, according to MYH11^{+}αSMA^{+} CAF IHF category. MYH11^{+}αSMA^{+} CAF phenotype is identified by IHF on the matched FFPE samples and then used to stratify samples.

CAF: cancer-associated fibroblasts; IQR: interquartile range.

Clinical characteristic		N (%)
Age - yr	Median (range)	63.8 (30.0 - 92.0)
Age group	18-65	278 (54.6)
	>65	231 (45.4)
Sex	Female	168 (33.0)
	Male	341 (67.0)
Histotype	Adenocarcinoma	393 (77.2)
	Squamous cell carcinoma	107 (21.0)
	Others*	9 (1.8)
Sample type	Needle biopsy	410 (80.6)
	Surgical resection	99 (19.4)
Performance status	0-1	404 (79.4)
	≥ 2	105 (20.6)
Prior line of treatment	0	148 (29.1)
	≥ 1	308 (60.5)
	NA	53 (20.4)
Regimen	ICI only	432 (84.9)
	ICI-CT combination	77 (15.1)
TPS	Negative	261 (51.2)
	1-49%	126 (24.8)
	≥ 50%	114 (22.4)
	NE	8 (1.6)
* Others: adenosquamous, neuroendocrine, sarcomatoid and undifferenciated carcinoma.		
Abbreviations: CT: chemotherapy; ICI: immune checkpoint inhibitors; NA: not available; NE: not evaluable; TPS: tumor proportion score.		

Table S1. Baseline characteristics of NSCLC patients (N=509). Related to Figure 1, Figure S1, Figure S2 and Table 1.

	OR group			PD group		
Clinical characteristic	P1	P2	P3	P4	P5	P6
Age	63	70	74	69	69	56
Sex	Male	Male	Male	Male	Male	Female
Histotype	Adenocarcinoma	Adenocarcinoma	Adenocarcinoma	Adenocarcinoma	Adenocarcinoma	Adenocarcinoma
Performance status	0	0	1	0	1	1
Prior line	1	5	2	2	1	2
Sample site	Lung	Adrenal	Lung	Lung	Lung	Lung
TPS (PD-L1, %)	10	0	0	0	95	0
Molecule	Nivolumab	Nivolumab	Nivolumab	Nivolumab	Pembrolizumab	Nivolumab
PFS	50.0	10.8	23.3	1.1	2	1.3
OS	86.8	36.4	37.3	4.7	11.7	10.0
Abbreviations: OR: objective response; OS; overall survival; PD: progressive disease; PFS: progression-free survival; TPS: tumor proportion score.						

Table S2. Clinical characteristics of patients with mTLS-positive NSCLC of the spatial transcriptomic experiment. Related to Figure 2, Figure S3, Table S3 and Table S4.

		ROI			Nuclei per AOI		
		TLS	Tumor	Stroma	TLS	Tumor	Stroma
OR group	P1	10	6	6	10614	9505	6083
	P2	10	6	6	19207	10784	9849
	P3	10	6	6	15439	10649	11252
PD group	P4	9	6	6	5828	9147	11810
	P5	10	6	6	8129	11035	8721
	P6	10	6	6	10381	12121	7707
Abbreviations: AOI: area of interest; mTLS: mature tertiary lymphoid structure; OR: objective response; PD: progressive disease; ROI: region of interest.							

Table S3. Number of ROI and nuclei per AOI for each patients with TLS-positive NSCLC of the spatial transcriptomic experiment. Related to Figure 2, Figure S3, Table S2 and Table S4.

Reference	https://doi.org/10.3390/cancers14051290	https://doi.org/10.1038/s41467-019-12464-3	https://doi.org/10.1038/s42003-022-04356-y	https://doi.org/10.1172/jci128672	https://doi.org/10.1016/j.immuni.2016.10.021	https://doi.org/10.1186/s13059-015-0620-6
Name	Schroeder 2022	Szabo 2019	Devi-Marulkar 2023	Freeman 2020	DeSimone 2016	Angelova 2015
List of genes	CD2	PMCH	FOXP3	ICOS	ADORA2A	CCL19
	CD247	FOXP3	TNFRSF9	CTLA4	TNFRSF18	CD34
	CD27	TIGIT	BTLA	TIGIT	LAG3	CD72
	CD28	CTLA4	TNFRSF4	CD28	LGALS9	CTLA4
	CD3D	IL2RA	PDCD1	TNFSF8	TNFRSF8	FOXP3
	CD3E	TNFRSF4	CD276	CD27	CD44	GADD45B
	CD3G	TNFRSF18	LAG3	CD226	TNFRSF4	GEM
	CD4	SELL	CCL20	TNFRSF9	PDCD1	IL1RL1
	CD5	ID3	CCL22	CD274	TNFRSF9	IL9R
	CD6	IFIT3	CXCL5	HAVCR2	ICOS	MADCAM1
	CD70	IFI6	CXCR3	CEACAM1	CTLA4	MYH10
	CD96	MAF	TNF	ENTPD1	TNFRSF14	NCF2
	CTLA4	STAT1	STAT4	LAG3	LAIR1	RCSD1
	DPP4	MAL	FOXA1	TNFRSF4	HAVCR2	RYR1
	FOXP3	LGALS3	IL10	TNFRSF18	TNFSF14	SELE
	ICOS	CCR7	ENTPD1	CD80	CD27	SELP
	IL2RA	PDZD8	LRRC32	BTLA	CD86	SFRP1
	IL2RB	CXCR6		CD40LG	CD70	SIT1
	LCK	AQP3			CD200R1	TIGIT
	LTB	SOCS1			TNFSF4	TLR10
	SH2D1A	MIR155HG			C10orf54	TLR2
	TRAT1	ANXA2			TIGIT	TLR7
	ZAP70	PSAT1			CD80	TLR8
		WARS			TNFRSF25	TRAF1
					CD40LG	WIPF1
					CD28	TGFB1
					IDO2	
					CD244	
					TMIGD2	

Table S7. RNAseq signatures of regulatory T cells pathway. Related to Figure 5 and Table S8.
